# Trans-provincial health impacts of atmospheric mercury emissions in China

**DOI:** 10.1038/s41467-019-09080-6

**Published:** 2019-04-02

**Authors:** Long Chen, Sai Liang, Maodian Liu, Yujun Yi, Zhifu Mi, Yanxu Zhang, Yumeng Li, Jianchuan Qi, Jing Meng, Xi Tang, Haoran Zhang, Yindong Tong, Wei Zhang, Xuejun Wang, Jiong Shu, Zhifeng Yang

**Affiliations:** 1 Key Laboratory of Geographic Information Science (Ministry of Education), School of Geographic Sciences, East China Normal University, Shanghai, 200241 People’s Republic of China; 2 State Key Joint Laboratory of Environment Simulation and Pollution Control, School of Environment, Beijing Normal University, Beijing, 100875 People’s Republic of China; 3 Ministry of Education Laboratory of Earth Surface Process, College of Urban and Environmental Sciences, Peking University, Beijing, 100871 People’s Republic of China; 4 The Bartlett School of Construction and Project Management, University College London, London, WC1E 7HB UK; 5 School of Atmospheric Sciences, Nanjing University, Nanjing, Jiangsu 210023 People’s Republic of China; 6 Department of Politics and International Studies, University of Cambridge, Cambridge, CB3 9DT UK; 7 School of Environmental Science and Engineering, Tianjin University, Tianjin, 300072 People’s Republic of China; 8 School of Environment and Natural Resources, Renmin University of China, Beijing, 100872 People’s Republic of China; 9 Institute of Environmental and Ecological Engineering, Guangdong University of Technology, Guangzhou, Guangdong 510006 People’s Republic of China

## Abstract

Mercury (Hg) exposure poses substantial risks to human health. Investigating a longer chain from economic activities to human health can reveal the sources and critical processes of Hg-related health risks. Thus, we develop a more comprehensive assessment method which is applied to mainland China—the largest global Hg emitter. We present a map of Hg-related health risks in China and estimate that 0.14 points of per-foetus intelligence quotient (IQ) decrements and 7,360 deaths from fatal heart attacks are related to the intake of methylmercury in 2010. This study, for the first time, reveals the significant impacts of interprovincial trade on Hg-related health risks across the whole country. For instance, interprovincial trade induced by final consumption prevents 0.39 × 10^−2^ points for per-foetus IQ decrements and 194 deaths from fatal heart attacks. These findings highlight the importance of policy decisions in different stages of economic supply chains to reduce Hg-related health risks.

## Abstract (web summary)

Mercury (Hg) is a global neurotoxic pollutant and has a long chain from economic activities to human health risks. Here the authors presented a map of Hg-related health risks in China and found significant impacts of interprovincial trade on health risks, such as the prevention of deaths from fatal heart attacks by the trade induced by final consumption.

## Introduction

Mercury (Hg) is a global neurotoxic pollutant, is persistent, and can be transported globally^1,2^. The methylation and bioaccumulation of inorganic Hg (IHg) in food webs can produce methylmercury (MeHg), a highly toxic form of Hg. MeHg exposure can cause neurocognitive deficits in foetuses and might be associated with cardiovascular effects in adults^3–5^. Since the middle of the twentieth century, MeHg exposure has caused considerable damage to human health. Minamata disease was first discovered in Japan in 1956 and has aroused global concern; approximately 3000 victims had been officially certified, 1784 of whom had already died as of 2001^6^. During 1971–1972, approximately 6000 people were poisoned, 452 of whom died, in the Poisonous Seeds Event (due to the consumption of organomercury-dressed seed) that occurred in Iraq^7^. Currently, the attributable societal costs of intelligence quotient (IQ) decrements caused by MeHg exposure are $16 billion in the United States and European Union^8,9^. Moreover, a tenfold increase in Hg levels in upper trophic level marine animals has been observed over the past ~150 years in the Arctic, where indigenous people rely heavily on marine-based diets^10,11^. To prevent these types of tragedies, the Minamata Convention, which was signed by over 100 nations, entered into force in 2017 to reduce global Hg-related health risks.

Hg-related health risks involve economic activities (e.g., labour and capital inputs, production activities, and consumption activities)^2,12^, environmental processes (e.g., atmospheric transport and deposition)^1,13^, food web transfers (e.g., bioaccumulation)^14,15^, and environmental health threats (e.g., MeHg intake and exposure dose–response)^4,16^. These processes form the chain of the biogeochemical Hg cycle. Previous studies have evaluated several specific components of this chain. For instance, studies found that atmospheric Hg emissions were driven by socioeconomic factors^12,17–19^, Hg deposition over one area could be substantially affected by the physical transport of Hg discharged from distant regions^20–22^, and MeHg intake in one area could originate from various types of foods in different global regions (e.g., seafood, rice, and freshwater fish)^23–25^. Giang and Selin^26^ developed a method to evaluate the impacts of changes in atmospheric Hg emissions on human health in the United States, which has the longest chain of the biogeochemical Hg cycle thus far. However, a longer chain of the biogeochemical Hg cycle from economic activities to human health has not been characterized. The limitations of biogeochemical Hg cycle chains in previous studies prevented them from identifying the impacts of interregional trade on Hg-related health risks and then proposing effective policy decisions to reduce these risks through interregional cooperation.

In this study, we develop a more comprehensive assessment method, for the first time, with which to investigate a longer chain of the biogeochemical Hg cycle from economic activities to human health. The investigation of this longer chain can reveal the sources and critical processes of Hg-related health risks under different stages of economic supply chains (e.g., primary inputs, production, and final consumption) and physical processes of Hg movement, which can support diverse Hg control measures to effectively reduce Hg-related health risks. This investigation can also reveal the considerable impacts of interregional trade on Hg-related health risks, highlighting regional pairs/groups that can collaboratively reduce Hg-related health risks of the whole system.

We use mainland China (hereafter referred to as China) as a case example in this study because people in China have experienced severe Hg-related health effects. China is the largest Hg emitter in the world, accounting for 33% (647 Mg yr^−1^) of the global anthropogenic Hg emissions to the atmosphere every year^27,28^. Anthropogenic Hg emissions in China have continuously increased in recent decades^28–30^. Increasing Hg emissions have resulted in large amounts of Hg deposition and outflows via atmospheric movement^31,32^. Hg deposition over China has led to severe health risks to exposed populations through the intake of various Hg-containing foods (e.g., rice, vegetables, pork, poultry, and fish), especially in Hg mining areas and cities^23,33,34^. Thus, the mitigation of Hg-related health risks is urgent in China, and such efforts would make a substantial contribution to the reduction of Hg globally. However, a longer chain of the biogeochemical Hg cycle from economic activities to human health in China has not been characterized.

To investigate a longer chain of the biogeochemical Hg cycle in China, this study develops a more comprehensive assessment method called the China Mercury Risk Source-Tracking Model (CMSTM) to track the biogeochemical Hg cycle in the context of physical and virtual transboundary flows (see Methods for details). Based on an intake inventory of MeHg and the dose–response relationship between MeHg intake and human health impacts, we present a map of Hg-related health risks (i.e., per-foetus IQ decrements and fatal heart attacks) in China for the first time. Based on a multiregional input–output (MRIO) model and atmospheric transport model, we distinguish the geographical and sectoral sources (e.g., primary suppliers, direct emitters, and final consumers) of the health risks for the population in each Chinese province and evaluate the impacts of interprovincial trade on the health risks in China. The findings of this study can support policy decisions in different stages of economic supply chains to effectively reduce Hg-related health risks.

## Results

### Hg-related health risks in China

The intake of MeHg results in 0.14 points of per-foetus IQ decrements and 7360 deaths from fatal heart attacks in China in 2010. Approximately 60.8% (0.08 points) of IQ decrements and 61.8% (4532 deaths) of fatal heart attacks are attributed to Chinese anthropogenic sources (hereafter referred to as attributable health risks). The remaining numbers are related to emissions from natural processes (e.g., oceanic evasions, volcanic eruptions, and crustal weathering) and foreign anthropogenic sources (see Supplementary Fig. 1 for provincial details).

**Fig. 1:**
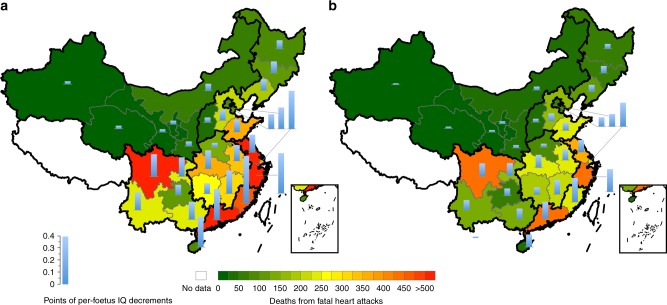
Spatial distributions of per-foetus intelligence quotient (IQ) decrements and fatal heart attacks induced by the intake of methylmercury (MeHg) in China. **a** Total number induced by the intake of MeHg from all mercury (Hg) emission sources, and **b** the number induced by the intake of MeHg from Chinese anthropogenic Hg emissions. The coloured background represents the deaths from fatal heart attacks, and blue bars represent the points of per-foetus IQ decrements

We observe differences in the spatial distributions of health risks among Chinese provinces (Fig. 1). For IQ decrements, Fujian ranks first, with 0.39 points of per-foetus IQ decrements, followed by Zhejiang (0.33 points) and Shanghai (0.32 points). The highest risk in Fujian is mainly caused by its high intake level of fishes (70.8 g capital^−1^ day^−1^) with high MeHg concentrations. In terms of fatal heart attacks, Guangdong ranks first, with 797 deaths, followed by Zhejiang (786 deaths) and Sichuan (749 deaths). The high risks in these provinces are attributed to high MeHg intake and a high incidence of heart attacks in the large population (Supplementary Fig. 2 and Data 1). In general, the numbers of IQ decrements and fatal heart attacks in China increase from north-western inland provinces towards south-eastern coastal provinces.

### Source identification and trans-provincial transport

**Fig. 2:**
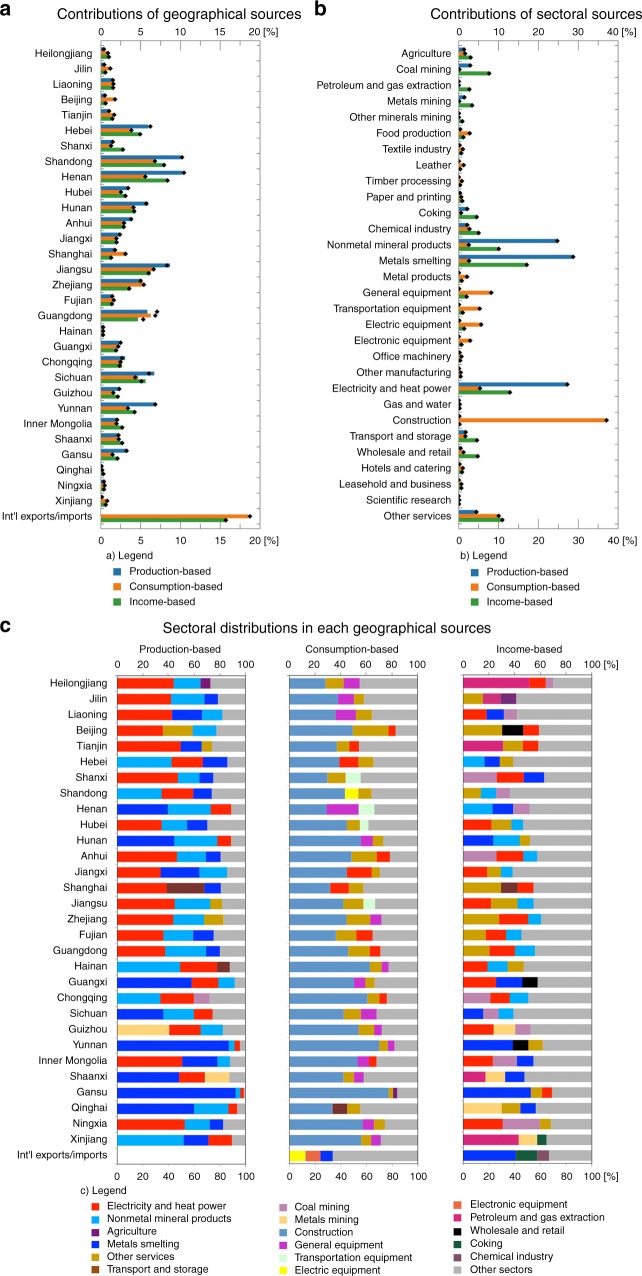
Contributions of anthropogenic sources to the national attributable per-foetus intelligence quotient (IQ) decrements (0.08 points) and fatal heart attacks (4532 deaths) in China. The attributable IQ decrements and fatal heart attacks refer to these provincial health risks caused by Chinese anthropogenic sources. **a**, **b** The results for fatal heart attacks and IQ decrements are shown as coloured bars and black solid diamonds, respectively. **c** The top three sectors are listed for each geographical source, and the remaining sectors are grouped as other sectors. The results for top sectors are the same for fatal heart attacks and IQ decrements. Int’l exports and Int’l imports represent international exports and international imports, respectively. The sector names are shorthand versions of the standard names (see Supplementary Table 1). Detailed results for the other sectors in **c** are shown in Supplementary Data 2

Considering the focus of policy decisions to effectively reduce Hg-related health risks in different stages of economic supply chains, we distinguish the geographical and sectoral sources of attributable health risks that represent the primary suppliers, direct emitters, and final consumers for the entire population in China. Source identification from the production, consumption, and income perspectives investigates the role of a source as a direct emitter, final consumer, and primary supplier, respectively. Such as investigation helps policy decisions regarding health risks caused by direct Hg emissions, induced by final consumption, and enabled by primary inputs (e.g., labour forces and capital). Figure 2 illustrates the contributions of various anthropogenic sources to the national attributable per-foetus IQ decrements (0.08 points) and fatal heart attacks (4532 deaths) in China. The production, consumption, and income perspectives reveal different distribution patterns of various geographical sources and sectoral sources. Henan, Shandong, and Jiangsu are major direct contributors to these national attributable health risks (Fig. 2a). Zhejiang, Shanghai, and Beijing have higher consumption-based health risks than production-based and income-based health risks, and Shanxi, Inner Mongolia, and Shaanxi are more important as primary suppliers than as direct emitters and final consumers.

In terms of sectoral sources (Fig. 2b), the smelting and pressing of metals, production and supply of electricity and heat power, and nonmetal mineral products sectors are the primary direct contributors to national attributable health risks, accounting for approximately 80.7%. From the consumption perspective, the final demand for products from the construction, general and special equipment, and electric equipment and machinery sectors mainly contributes to the national risks. In particular, the final demand for products from the construction sector is the largest contributor because rapid industrialization and urbanization require substantial construction activities. From the income perspective, the primary inputs of sectors, such as coal mining and dressing, petroleum and natural gas extraction, metals mining and dressing, and wholesale and retail, are important contributors.

For each geographical source, we investigate the top three sectors from the production, consumption, and income perspectives, and the results indicate different distribution patterns (Fig. 2c). From the production perspective, the production and supply of electricity and heat power sector is the most important sector for most geographical sources. However, the most important sectors are the nonmetal mineral products sector in Hebei, Shandong, and Hainan and the smelting and pressing of metals sector in Henan, Guangxi, and Yunnan. From the consumption perspective, the construction sector is the most important sector for all geographical sources. From the income perspective, the top three sectors vary among different geographical sources.

**Fig. 3:**
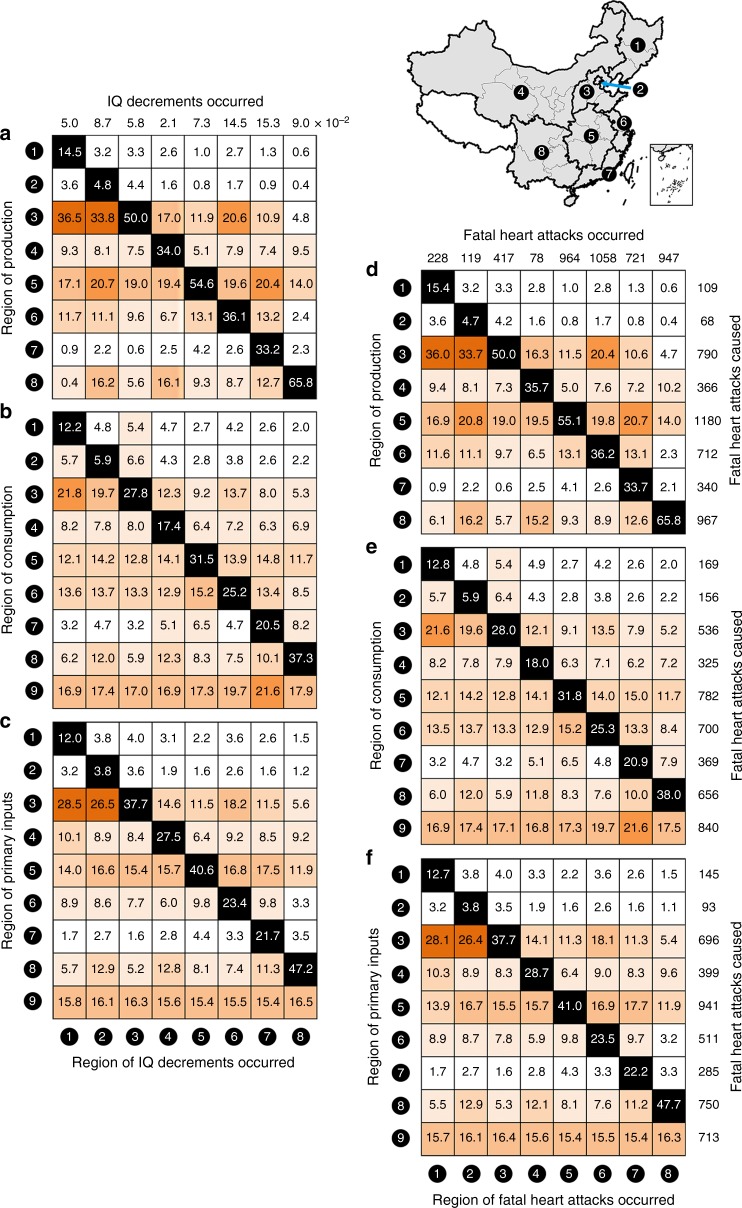
Composition of attributable per-foetus intelligence quotient (IQ) decrements and fatal heart attacks in a given Chinese region. The composition in a given region results from anthropogenic mercury (Hg) emissions discharged by production activities (**a**, **d**), induced by final consumption (**b**, **e**), and enabled by primary inputs (**c**, **f**) of local and external regions. **a**–**c** The results for IQ decrements and **d**–**f** the results for fatal heart attacks. Each cell in the grid shows the fraction of the health risk (%) of the column region caused by the production, final consumption, and primary inputs of the row region. The total points of per-foetus IQ decrements that occur in each region are shown at the top of **a**, and the total deaths from fatal heart attacks that occur in each region are shown at the top of **d**. The total deaths from fatal heart attacks caused by each region are also shown on the right of **d**–**f**. Regions 1 to 9 represent the Northeast, Beijing-Tianjin region, North, Northwest, Central, Central Coast, South Coast, Southwest, and Int’l exports/imports (see Supplementary Fig. 3 for regional definitions). A black-coloured cell in a region reflects the fraction of health risks in the region caused by local activities. In other cells, darker orange cells indicate higher fractions

Based on the source identification of the national Hg-related health risks, we further distinguish sources of attributable health risks for each geographical receptor in China, illustrating trans-provincial transport of Hg-related health risks in China via physical and virtual Hg transport (Fig. 3). Figure 3a, d shows the percentage of attributable IQ decrements and fatal heart attacks, respectively, in a region caused by the physical transport of Hg emitted from local and external production activities. High trans-provincial health risks occur from emitters such as the North and Central regions. For example, 36.5% and 33.8% of the attributable IQ decrements in the Northeast and the Beijing-Tianjin region originate from the North, respectively. On average, 55.1% of the attributable IQ decrements and 54.6% of the attributable fatal heart attacks are caused by the physical transport of Hg emitted in a different region.

Figure 3b, c, e, f shows the percentage of attributable health risks in a region caused by Hg emissions induced by local and external final consumption (Fig. 3b, e) and enabled by local and external primary inputs (Fig. 3c, f), involving virtual transport via trade and physical transport via atmospheric movement. In addition to physical transport, virtual transport via trade allows the health risks to be more widely scattered around the nation, promoting the trans-provincial transport of Hg-related health risks in China. For example, nationally, 3286 deaths and 3064 deaths from fatal heart attacks are induced by external final consumption and external primary inputs, respectively, and these values are even larger than the individual effect of physical Hg transport (2473 deaths).

Regionally, the contribution of final consumption in the Beijing-Tianjin region, Central Coast, and South Coast to health risks that occur in other regions (Fig. 3b, e) is larger than the individual effect of physical Hg transport (Fig. 3a, d), because these regions are in the downstream stages of the economic supply chains in China and mainly serve as final consumers of goods and services from upstream suppliers in other regions. Similarly, the contribution of primary inputs in the Northeast and Northwest to health risks occurring in other regions (Fig. 3c, f) is larger than the individual effect of physical Hg transport (Fig. 3a, d). These regions are in the upstream stages of the economic supply chains in China and mainly serve as resource suppliers to downstream users in other regions. Moreover, we further explore the flows of Hg-related health risks at the provincial scale (shown in Supplementary Data 3). In general, trans-provincial transport of Hg-related health risks in China shows a different spatial distribution from the production, consumption, and income perspectives.

### Impacts of interprovincial trade

**Fig. 4:**
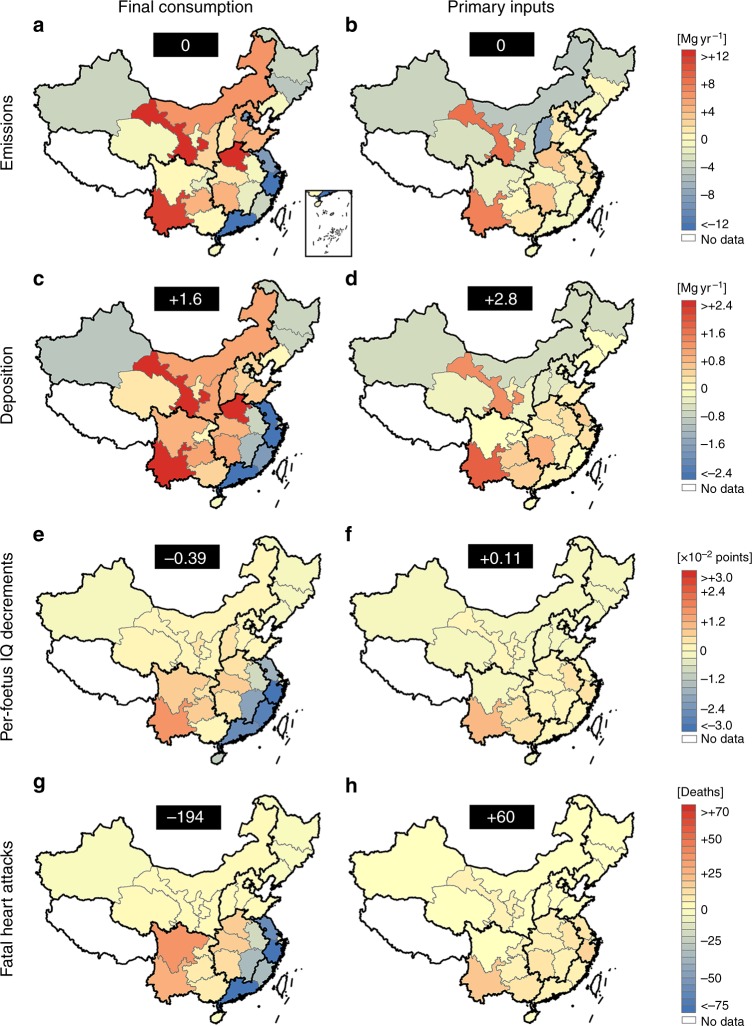
Spatial relocation of atmospheric mercury (Hg) emissions and deposition as well as Hg-related intelligence quotient (IQ) decrements and fatal heart attacks over China due to interprovincial trade. The relocation is evaluated by the difference between interprovincial trade of products versus producing the same products locally under a hypothetical absence of interprovincial trade. **a**, **c**, **e**, **g** The results for the interprovincial trade of products induced by final consumption, and **b**, **d**, **f**, **h** the results for the interprovincial trade of products enabled by primary inputs. The number at the top of each panel shows the net national change with the sum of changes in all provinces

The interprovincial trade of goods and services partly separates the locations of final consumption and primary inputs from the locations of direct Hg emissions and serves as the greatest feature for the final consumers and primary suppliers in economic supply chains. We set up a hypothetical scenario with an absence of interprovincial trade for comparison with the existence of interprovincial trade to evaluate the impacts of interprovincial trade on Hg-related health risks in China from the consumption and income perspectives. We assume that the trade partners can produce the same goods and services, which are originally involved in interprovincial trade locally. Figure 4 shows the impacts of interprovincial trade on atmospheric Hg emissions and deposition as well as Hg-related IQ decrements and fatal heart attacks over China. Interprovincial trade induced by final consumption contributes to the flow of emissions and deposition from the southeast coast to inland regions, resulting in the prevention of Hg-related health risks along the southeast coast and additional Hg-related health risks in inland regions. Interprovincial trade enabled by primary inputs contributes to the flow of emissions and deposition from the northern regions to the southern regions, resulting in the prevention of Hg-related health risks in the northern regions and additional Hg-related health risks in the southern regions. For example, 99 deaths from fatal heart attacks are prevented in Zhejiang due to exports of emissions through interprovincial trade, whereas an additional 35 deaths occur in Sichuan due to imports of emissions through interprovincial trade (Fig. 4g).

We sum up changes in all provinces and evaluate the net impacts of interprovincial trade over the whole of China. We find that interprovincial trade relocates the spatial distribution of atmospheric Hg emissions over China but does not change the total Hg emissions of the whole country. Similarly, interprovincial trade relocates the spatial distribution of atmospheric Hg deposition over China but shows ignorable impacts on the gross Hg deposition of the whole country. However, interprovincial trade induced by final consumption enables the whole country to avoid 0.39 × 10^−2^ points of per-foetus IQ decrements and 194 deaths from fatal heart attacks (Fig. 4g). Interprovincial trade enabled by primary inputs induces an additional 0.11 × 10^−2^ points of per-foetus IQ decrements and 60 deaths from fatal heart attacks (Fig. 4h).

Although the net impact of atmospheric Hg deposition on the whole country is less obvious, the changes in the spatial distributions of the deposition induce a regional asymmetry of changes in MeHg concentrations in food products and subsequent MeHg intake over different deposited regions. The ingestion of marine fish, freshwater fish, and rice, which contain high MeHg concentrations, mainly contributes to the intake of MeHg for Chinese populations (see Supplementary Data 4). These food products are mainly harvested in marine-fishing, aquaculture, and rice-growing areas that are located in the eastern and southern lands and seas of China (i.e., sensitive areas). The same amount of atmospheric Hg deposition over these areas can cause a greater impact on the intake of MeHg for Chinese populations than that over inland regions. Interprovincial trade induced by final consumption contributes to the transfer of Hg from sensitive areas (e.g., the southeast coast) to less sensitive areas (e.g., inland regions), while interprovincial trade enabled by primary inputs contributes to the transfer of Hg from less sensitive areas (e.g., northern regions) to sensitive areas (e.g., southern regions) (Fig. 4). The net impact of interprovincial trade on Hg-related health risks across the whole country is attributed to these transfers of Hg.

Previous studies, which investigated a shorter chain of the biogeochemical Hg cycle from economic activities to atmospheric Hg emissions and deposition, illustrated the spatial relocations of atmospheric Hg emissions and deposition over China, but showed ignorable net impacts of interprovincial trade on the Hg pollution over the whole of China^12,17,18,35^. In this study, we reveal that interprovincial trade not only changes the spatial distribution of Hg pollution over China but also considerably changes the Hg-related health risks across the whole country. Compared to previous studies, this finding can support Hg-related policy making of the Chinese government to achieve a national goal of Hg reduction.

## Discussion

Interprovincial trade considerably changes the spatial distribution of the Hg-related health risks in China, reinforcing the importance of source identification and relevant policy decisions in different stages of economic supply chains. The longer chain of the biogeochemical Hg cycle described in this study first provides a map of Hg-related health risks in China and identifies the sources of the risks for primary suppliers, direct emitters, and final consumers. This study can provide scientific foundations for policy decisions in different stages of economic supply chains (i.e., production-side, demand-side, and supply-side controls) to effectively reduce Hg-related health risks. Production-side control measures, such as improving energy use efficiency and establishing Hg removal facilities, are effective in critical geographical and sectoral sources whose direct Hg emissions can induce high health risks. For example, the measures are effective for sources such as nonmetal mineral products in Shandong, smelting and pressing of metals in Henan, and production and supply of electricity and heat power in Jiangsu. Demand-side control measures, such as influencing consumption behaviours through changing consumption taxes, are effective in critical geographical and sectoral sources the final consumption of which drives high upstream health risks. For example, the consumption of products from the construction, general and special equipment, and electric equipment and machinery sectors is preferentially concerned with the demand-side measures. Developed regions, such as the Beijing–Tianjin region, Central Coast, and South Coast, are prior to implement the demand-side control measures (Fig. 3). Supply-side control measures are effective in critical geographical and sectoral sources, primary inputs of which enable high downstream health risks. Decision makers could choose to invest in dominant enterprises of geographical and sectoral sources with less income-based health risks and limit loans and subsidies to these sources with high income-based health risks (e.g., smelting and pressing of metals and coal mining and dressing sectors). The effective measures are different for sources located in different stages of economic supply chains. For example, the production-side control measures are more effective than other measures in Henan, Shandong, and Jiangsu, while the demand-side control measures are more effective than other measures in Zhejiang, Shanghai, and Beijing. For Shanxi, Inner Mongolia, and Shaanxi, the supply-side control measures are more effective than other measures.

The spatial relocation of Hg-related health risks due to interprovincial trade highlights regional pairs/groups that can collaboratively reduce Hg-related health risks of the whole system. Final consumers such as Shanghai, Zhejiang, and Guangdong could transfer related technologies and capital to their upstream direct emitters (e.g., Henan and Gansu) to reduce their Hg-related health risks. Primary suppliers such as Shanxi and Inner Mongolia could transfer related technologies and capital to their downstream users (e.g., Gansu and Yunnan) to reduce their Hg-related health risks.

The Chinese government has been promoting the development of urban agglomerations in recent years. Urban agglomerations are typically major importers and consumers of products. Their rapid development will facilitate the interregional flows of products in China. The intensified interprovincial trade will further affect Hg-related health risks for the Chinese population. To reduce Hg-related health risks caused by interprovincial trade, it is vital to take measures (discussed above) at different stages of economic supply chains.

Investigating a longer chain of the biogeochemical Hg cycle from economic activities to human health in this study provides more scientific foundations for the reduction of Hg-related health risks than previous studies investigating a shorter chain of the biogeochemical Hg cycle from economic activities to atmospheric Hg emissions and deposition^12,17,18,35^. First, investigating a longer chain can provide a map of Hg-related health risks in China for the first time. The ultimate goal of Hg reductions in the Minamata Convention is to reduce Hg-related health risks to human beings. The investigation of atmospheric Hg emissions and deposition conducted in previous studies cannot fully support policy decisions to achieve this ultimate goal. Second, the shorter chain of the biogeochemical Hg cycle investigated in previous studies illustrates the spatial relocations of atmospheric Hg emissions and deposition over China resulting from interprovincial trade. However, this perspective shows ignorable net impacts of interprovincial trade on the Hg pollution over the whole of China. Investigating a longer chain in this study, for the first time, reveals the considerable net impacts of interprovincial trade on Hg-related health risks over China. Third, this study focusses on Hg-related health risk control and presents more targeted results than previous studies. For instance, the top contributor shifts from the consumption by Shandong and Guangdong to the consumption by Jiangsu, with the transition of endpoints from atmospheric Hg emissions and deposition to Hg-related health risks (see Supplementary Data 5). More details on the comparisons between this study and previous studies are provided in the Supplementary Information.

Our method and associated results are subject to certain uncertainties and limitations. A Monte Carlo simulation with 10,000 samplings is conducted on variables in different steps of our analyses, including the compilation of Hg emission inventory, the MRIO analyses, the simulation of chemical transport model, the compilation of intake inventory of MeHg, and the evaluation of human health impacts. The P10 and P90 values of the statistical distributions are set as the lower and upper limits of the uncertainty range. A detailed description of uncertainties is presented in the Supplementary Information. In summary, nationally, the uncertainties of the points of per-foetus IQ decrements and the deaths from fatal heart attacks are estimated at (0.03, 0.25) and (2992, 15,383), respectively. Meanwhile, the uncertainties of changes in per-foetus IQ decrements are estimated at (−0.77, −0.07) for interprovincial trade induced by final consumption and (0.02, 0.19) for interprovincial trade enabled by primary inputs. Those for fatal heart attacks are estimated at (−412, −76) for interprovincial trade induced by final consumption and (25, 112) for interprovincial trade enabled by primary inputs. The uncertainties in our results vary among regions, with greater uncertainties observed for regions with less data.

We have not accounted for the impacts of aquatic Hg discharges and accurate lag time in the response between Hg emissions and exposure risks in this study. Additional measurements are needed for food products from polluted soils and waters and the associated food circulation in markets in the future. Meanwhile, collecting annual data regarding Hg concentrations of food products and determining accurate lag times would improve the accuracy of our results.

In addition to economic supply chains and Hg emission sources, the biogeochemical Hg cycle and related adverse health impacts are also influenced by multiple extrinsic and intrinsic factors (see Eagles-Smith et al.^36^ and Obrist et al.^37^). This study mainly focusses on the integration of economic supply chains with Hg-related health impacts. Moreover, the mechanisms between many extrinsic and intrinsic factors and Hg cycle still remain inconsistent or unknown. Thus, the CMSTM in this study has not considered these factors (see Supplementary Information). However, these factors can be practicably incorporated into the CMSTM, when future studies can clearly characterize their dynamics, mechanisms, and modelling methods. Understanding these factors and related mechanisms would support the proper evaluation of the effectiveness of Hg reduction activities on human health risks and the future implementation of Hg pollution control policies such as the Minamata Convention.

In general, our database and the CMSTM can be updated and improved as better statistical data, monitoring data, and mechanisms become available in the future. With these improvements, the framework of the CMSTM could be applied to other contaminants that also circulate in various environmental media at different spatial scales in the context of rapid global changes.

## Methods

### Framework of the CMSTM

In this study, a more comprehensive assessment method is developed to investigate the chain of the biogeochemical Hg cycle from economic activities to human health. This new method is called CMSTM and tracks the biogeochemical cycle of Hg emitted from Chinese anthropogenic sources and relevant Hg-related health risks in China.

Supplementary Figure 4 illustrates the framework of the CMSTM. The model consists of seven components: a Hg emission inventory of Chinese anthropogenic sources, an atmospheric transport model, changes in food MeHg resulting from atmospheric deposition, a human intake inventory of MeHg, human health impacts due to intake exposure, Hg emissions from natural sources and foreign anthropogenic sources, and imports of food products from foreign countries. The first five components occur over mainland China and the coastal seas, which are used to track the emission sources of Hg-related health risks in China. The boundaries for all 30 Chinese provinces and 8 regions are shown in Supplementary Fig. 3. Moreover, the latter two components occur in foreign countries or represent natural sources (e.g., oceanic evasions, volcanic eruptions, and crustal weathering), serving as two external components that are quantified as constant contributions. We illustrate detailed methods for these components in the following sections.

### Hg emission inventory

First, we evaluate production-based Hg emissions. Atmospheric Hg is mainly emitted from human production activities, such as fuel consumption, nonferrous metal smelting, production of building materials, primary Hg mining, and waste incineration. As a common practice, emissions can be calculated by multiplying the energy usage or product yields by the corresponding emission factors. We compiled a Chinese production-based Hg emission inventory in our previous work^35^, which is used as the satellite account of a MRIO model in this study. The production-based Hg emission inventory includes primary anthropogenic sources and secondary Hg emissions from the disposal of waste/by-products and is distributed as point and non-point sources.

Second, we compile consumption-based and income-based Hg emissions. MRIO models have been widely used in analysing environmental issues in the context of increasing interregional trade^38–41^. MRIO models are based on a multiregional input–output table that describes the product exchanges within and among regions. Treating production-based Hg emissions of sectors as the satellite account of a MRIO table, an environmentally extended MRIO (EE-MRIO) model can be constructed (Supplementary Tables 1 and 2). The EE-MRIO model tracks Hg emissions from the region of final consumption (i.e., final consumers) to the region of production (i.e., direct emitters) through product supply chains, and it also tracks Hg emissions from the region of primary inputs (i.e., primary suppliers) to the region of production (i.e., direct emitters) through product sale chains. We use the MRIO table developed by Liu et al.^42^ to evaluate consumption-based and income-based Hg emissions for China in 2010. A brief introduction to the MRIO model is provided below, and detailed descriptions are provided in previous studies^38,43^.

Region- and sector-specific production-based, consumption-based, and income-based Hg emissions can be measured using Eqs. 1, 2 and 3, respectively:

1 $${P}_r = {\mathbf{\psi }}\prime {\mathbf{X}}_r,$$

2 $${C}_r = {\mathbf{\psi }}\prime ({\mathbf{I}} - {\mathbf{A}})^{ - 1}{\mathbf{F}}_r,$$

3 $${S}_r = {\mathrm{V}}_r({\mathbf{I}} - {\mathbf{B}})^{ - 1}{\mathbf{\psi }},$$

where *P*_*r*_, *C*_*r*_, and *S*_*r*_ indicate region- and sector-specific production-based, consumption-based, and income-based Hg emissions, respectively. The column vector **ψ**, regarded as the direct emission intensity, represents Hg emissions according to a unitary output of sectors, which is equal to the Hg emissions of each sector divided by its total output. **X**_*r*_ indicates the total output of sector *r* or each sector in region *r*. The notation ‘'’ indicates the transposition of the vector **ψ**. The column vector **F**_*r*_ indicates the final demand of sector *r* or region *r*. The row vector **V**_*r*_ indicates the primary input in sector *r* or region *r*. **A** represents the direct input coefficient matrix, whose element a_*ij*_ shows direct purchases from sector *i* by the unitary output of sector *j*. **B** represents the direct output coefficient matrix, whose element b_*ij*_ shows direct sales from sector *i* to sector *j* in terms of unitary output in sector *i*. **A** and **B** can characterize interregional economic interactions between sectors. **I** denotes the identity matrix.

The matrix (**I** − **A**)^−1^ is the Leontief inverse matrix, which captures the effect of product supply chains by describing both direct and indirect inputs from various sectors to satisfy the unitary final demand of specific sectors. The matrix (**I** − **B**)^−1^ is the Ghosh inverse matrix, which captures the effect of product sale chains by describing both direct and indirect outputs from various sectors enabled by the unitary primary input of specific sectors. The results of the region-specific production-based, consumption-based, and income-based Hg emissions are shown in Supplementary Fig. 5.

A further application of these basic input–output formulas is the quantification of the emissions embodied in trade. For example, the Hg emissions in region *i* caused by the final demand of sector *j* or region *j* can be calculated as follows:

4 $${C}_{ij} = {\mathbf{\psi }} _i^\prime ({\mathbf{I}} - {\mathbf{A}})^{ - 1}{\mathbf{F}}_j.$$

The Hg emissions in region *i* enabled by primary inputs from sector *j* or region *j* can be calculated as follows:

5 $${S}_{ij} = {\mathrm{V}}_j({\mathbf{I}} - {\mathbf{B}})^{ - 1}{\mathbf{\psi }}_i,$$

where **ψ**_*i*_ denotes a vector with the direct emission intensity for province *i* but zero for other provinces, **F**_*j*_ indicates the final demand of sector *j* or region *j*, and **V**_*j*_ indicates the primary input in sector *j* or region *j*.

### Atmospheric transport model

The GEOS-Chem chemical transport model (version 9-02; http://geos-chem.org) is used to simulate the atmospheric Hg transport and deposition over China. The model tracks three Hg species in the atmosphere (i.e., Hg^0^, Hg^II^, and particulate Hg (Hg^P^)) and nearly all of the Hg is in the inorganic form. Moreover, Hg^0^ can be oxidized to Hg^II^ by Br atoms, Hg^II^ can be reduced to Hg^0^ through light in droplets, and equilibrium partitioning can occur between Hg^II^ and Hg^P^^13,44^. In addition to simulating Hg cycle in the atmosphere, the global three-dimensional atmosphere model is also coupled to two-dimensional surface ocean and terrestrial slab models to simulate the air–sea exchange and air–soil exchange of Hg^13,45^. Meteorology in the model is driven by the Goddard Earth Observing System (GEOS-5) assimilated fields taken from the NASA Global Modeling and Assimilation Office (GMAO). Global simulations from 2008 to 2010 are conducted at a 4° × 5° horizontal resolution with 47 vertical levels from the surface to 0.01 hPa. These simulations serve as boundary conditions for a nested, higher-resolution simulation at a horizontal resolution of 1/2° × 2/3° over China, which follows the method presented in our previous work^35^. The nested model was evaluated against a series of observations in the previous work. We run the nested model for the year 2010 with an initial spin-up of 3 months in 2009. Moreover, we implement the explicit tagging technique from our previous work^35^ to track the geographical and sectoral sources of atmospheric deposition over each Chinese province and sea. The explicit tagging technique is characterized by tagging the Hg emitted from a series of sources using additional model variables in a single model simulation.

Changes in Hg deposition are used as a measure of Hg enrichment to an ecosystem^21^ and changes in Hg concentrations in Hg-containing food products. We sum the deposition over all GEOS-Chem grid boxes for each of the 30 Chinese provinces and 4 seas (i.e., the Bohai Sea, Yellow Sea, East China Sea, and South China Sea) to evaluate the changes in Hg deposition and consequent Hg concentrations in food products in each specific region.

### Changes in food MeHg resulting from atmospheric deposition

According to previous studies^23,46^, the following 10 categories of Hg-containing food products are selected as the main intake pathways of total Hg (THg; THg = MeHg + IHg) and MeHg for the Chinese population in this study: rice, wheat, beans, vegetables, pork, poultry, milk, eggs, marine fish, and freshwater fish. Here, marine fish and freshwater fish include major fish species in China (e.g., grass carp, crucian carp, common carp, catfish, silver pomfret, and yellow croaker) and other aquatic products (e.g., shrimp, crab, and shellfish). Previous analyses and recent assessment data have shown that changes in MeHg in ecosystems are driven by THg availability in environmental media^47,48^, which has led to the assumption that MeHg concentrations in fish respond proportionally to changes in the atmospheric inputs of THg to environmental media^26^. The influence of atmospheric Hg deposition to rice has been a topic of debates in the literature^49–52^. A number of studies have suggested that atmospheric deposited Hg is more bioavailable for methylation and uptake by rice^49–51^, and illustrated a positive linear correlation between rice MeHg and bioavailable Hg from atmospheric deposition under the irrigation of drinking water^51,53^. In view of this, we assume that MeHg concentrations in rice also respond proportionally to changes in atmospheric Hg inputs in this study. We also apply this assumption to all other food products in this study due to data unavailability. Changes in MeHg concentrations in food products that are caused by changes in the atmospheric Hg deposition in a specific region are calculated using Eq. 6:

6 $$\frac{{{\mathrm{\Delta Dep}}_{ijk}}}{{{\mathrm{Dep}}_i}} = \frac{{{\Delta C}_{ijk}}}{{{C}_i}},$$

where ΔDep_*ijk*_ represents the changes in atmospheric deposition over region *i* when the emissions from geographical source *j* or sectoral source *k* are changed; Dep_*i*_ represents the gross deposition over region *i*; Δ*C*_*ijk*_ denotes the changes in MeHg concentration in a specific food product harvested in region *i* when the emissions from geographical source *j* or sectoral source *k* are changed; and *C*_*i*_ denotes the total MeHg concentration in a specific food product harvested in region *i*. Moreover, due to data unavailability for specific source regions of international imports of food products, the global regions outside of China are treated as a whole region in this study, and changes in MeHg concentrations in imported food products are related to changes in the atmospheric Hg deposition in the whole region.

The conversion of THg in environmental media to MeHg in food products is controlled by numerous ecosystem factors^15,36,54^. Therefore, the assumption of proportional responses in this study dramatically simplifies the real-world dynamics of Hg in ecosystems. However, we use the simplified assumption to isolate the effect of deposition on MeHg in food products while keeping other factors constant. In addition, due to unavailability of other assumptions, the assumption of a proportional response has been assumed both in this study and previous studies^26,55^.

### Intake inventory of MeHg

We compile an intake inventory of MeHg for the Chinese population at the provincial scale based on our previous study^46^. The method in our previous study was used to compile an intake inventory at the regional scale, such as the intake of MeHg for the population in Central China. We apply and update the method in this study and involve provincial information to compile an intake inventory at the provincial scale, in order to evaluate the trans-provincial health risk resulting from atmospheric Hg emissions. The method for compiling the inventory includes three steps: compiling MeHg concentrations in food products, modelling the trade of food products, and evaluating estimated daily intake (EDI). We briefly elaborate the method as follows and illustrate how provincial information is used to compile the provincial intake inventory.

First, we compile MeHg concentrations in food products harvested in each province. Concentrations of THg and MeHg in food products harvested in specific provinces are collected from previous studies conducted by authoritative scientific research institutions in China and published in peer-reviewed journals (see Supplementary Data 4). Some data are retrieved from our previous study^46^, but we classify the data from the regional scale to the provincial scale according to the location information from previous literature. The Chinese government has set limits for concentrations of THg or MeHg in food products (see Supplementary Data 4); thus, we assume that the food products whose THg or MeHg concentrations exceed the limits would not be allowed to circulate in markets and are hence not considered in our analysis. To address data unavailability for a specific food product in a province, we use the average data from other provinces in the same region to represent Hg concentrations for this province.

Compared to THg, data for MeHg concentrations are scarce in China. Previous studies have suggested that the changes in MeHg concentrations were driven by THg availability^47,48^, such as the significant linear correlation between THg and MeHg in fish^14^ and the logarithmic linear correlation between THg and MeHg in rice^56^. Here, following our previous studies^46,55^, we establish the best linear correlation between THg and MeHg concentrations for certain food products whose published data are abundant (*n* > 10), such as rice, vegetables, marine fish, and freshwater fish. For food products with limited data (i.e., beans, pork, poultry, and eggs), we average the ratios of MeHg to THg concentration using the limited paired data (see Supplementary Data 4). The average ratios are 0.35, 0.54, 0.55, and 0.46 for beans, pork, poultry, and eggs, respectively. We estimate MeHg concentrations for each food product harvested in each province based on THg concentrations. The figures of the linear correlation between MeHg concentrations (*C*_MeHg_, ngHg g^−1^) and THg concentrations (*C*_THg_, ngHg g^−1^) and associated equations are provided in Supplementary Fig. 6.

Second, we model the interprovincial trade of food products. MRIO models characterize the trade of goods and services, including the trade of agricultural products^17,38,39^. We use a MRIO model to simulate the interprovincial trade of food products in this study. The MRIO table for China is in 2010 and is developed by Liu et al.^42^. Monetary data of the final demand including urban household consumption, rural household consumption, and government consumption for a given province in the MRIO table are used to describe the sources of a specific food product consumed in the province. The final demand data for the farming, forestry, animal husbandry, and fishery sector from the MRIO table (see Supplementary Table 1) are used for the simulation of food trade.

To simulate the trade of each type of food products, we assume that the total final demand of the farming, forestry, animal husbandry, and fishery sector in a province consists of the final demand of all agricultural products that have linear relationships with the production of all agricultural products in the province. Thus, we introduce a ratio of its output value to gross agricultural output to extract the final demand data for the specific food product from the total final demand data of the farming, forestry, animal husbandry, and fishery sector in the MRIO table for each province. The output values of food products and gross agricultural output are from the China Agriculture Yearbook^57^. However, there are no direct output values for certain food products from the China Agriculture Yearbook^57^, such as rice, wheat, and beans. In this case, we multiply production volumes from the yearbook by prices from the China Yearbook of Agricultural Price Survey^58^ to obtain the output values. Meanwhile, for food products such as rice, wheat, beans, milk, and marine fish, we also introduce the values of international imports from the China Agriculture Yearbook^57^ and China Fisheries Yearbook^59^ to describe source contributions of the international imports. For the other food products (i.e., vegetables, pork, poultry, eggs, and freshwater fish), the international imports are not considered since the fraction of their imports is less than 1% of their total consumption volumes in 2010^60^. Due to data unavailability, we do not distinguish specific source regions outside of China for international imports and treat the global regions outside of China as a whole region. The detailed data for output values of each type of food products are provided in Supplementary Data 6. Moreover, for marine fish, we estimate the source contributions of four coastal seas to the coastal provinces based on statistics from the China Fisheries Yearbook^59^ (see Supplementary Table 3), which is not considered in our previous study^46^. The equations for the calculation of ratios are provided below:

7 $${S}_{ijk} = {F}_{jk} \times \frac{{{P}_{ik} + {T}_{ik}}}{{{\mathrm{GAP}}_k + \mathop {\sum}\nolimits_i {{\kern 1pt} {T}_{ik}} }},$$

8 $${\mathrm{Per}}_{ijk} = \frac{{{S}_{ijk}}}{{\mathop {\sum}\nolimits_k {{\kern 1pt} {S}_{ijk}} }},$$

where *F*_*jk*_ represents the final demand of the farming, forestry, animal husbandry, and fishery sector in province *j* driven by the supply of province *k*, which is derived from the Chinese MRIO table^42^; *S*_*ijk*_ represents the final demand of food *i* in province *j* driven by the supply of province *k*; *P*_*ik*_ represents the output values of food *i* in province *k*, which are taken from the China Agriculture Yearbook^57^; GAP_*k*_ represents the gross agricultural output of province *k*, which is taken from the China Agriculture Yearbook^57^; *T*_*ik*_ represents the international imports of food *i* (i.e., rice, wheat, beans, milk, and marine fish) in province *k* from the rest of world, and the rest of world is treated as a whole region due to data unavailability of specific source regions; and Per_*ijk*_ is the source contribution of food *i* in province *j* driven by the supply of province *k*.

The simulated trade of each type of food products among Chinese provinces is shown in Supplementary Fig. 7.

Third, we evaluate EDI of MeHg. By combining the MeHg concentrations in food products harvested in specific provinces and interprovincial trade of the food products, we can estimate the MeHg concentrations in food products consumed in specific provinces. Subsequently, we can evaluate the EDI of MeHg by multiplying the MeHg concentrations in consumed food products by the intake rate of each type of food products for the population in each province.

We obtain the intake rate of food products from the data of per-capita consumption. The provincial data of per-capita consumption for rural and urban populations are taken from the China Statistical Yearbook^61^ and provincial statistical yearbooks in 2011 (http://tongji.cnki.net/kns55/Dig/dig.aspx) (see Supplementary Data 7). The per-capita consumption amount (kg year^−1^ capita^−1^) for the rural population is taken directly from these yearbooks. For urban population, the per-capita consumption amount in each province can be estimated from the data on per-capita consumption expenditures (Yuan year^−1^ capita^−1^) in each province and total national consumption amounts, which can be directly obtained from these yearbooks (see Supplementary Data 7). Per-capita consumption is close to per-capita intake, but it is not the direct intake by humans and may slightly overestimate or underestimate the intake. Thus, we use the total national intake rates of food products to adjust the per-capita consumption for the population in each province. The total national intake rates of food products are taken from the China Health and Nutrition Survey (CHNS), which was conducted by the Bureau of Disease Control and Prevention in the National Health and Family Planning Commission during 2010−2013. The survey covered 205 monitoring sites in 31 provinces and monitored 250,000 population samples. The national intake rates of major food products (e.g., rice, wheat, vegetables, pork, poultry, and fish) were estimated in the survey and reported in an official publication (see Supplementary Data 7). Based on the national intake rates, we adjust the per-capita consumption to per-capita intake for the population in each province (see Supplementary Data 8). This database is currently the most comprehensive, unified, and reliable data sources, compared to the databases used in our previous studies^46,55^.

The equations for the calculation of EDI are provided below:

9 $${I}_{ij} = {\mathrm{CON}}_{ij} \times \frac{{{\mathrm{NI}}_i}}{{{\mathrm{NCON}}_i}},$$

10 $${\mathrm{EDI}}_j = \mathop {\sum}\limits_{ik} {{\kern 1pt} ({\mathrm{Per}}_{ijk} \times {I}_{ij} \times {C}_{ik})/{\mathrm{BW}}},$$

where CON_*ij*_ represents the per-capita consumption amount (g d^−1^ capita^−1^) of food *i* by the population in province *j* and NCON_*i*_ represents the per-capita consumption amount of food *i* nationally. NI_*i*_ indicates the per-capita intake rate of food *i* nationally, and *I*_*ij*_ indicates per-capita intake rate (g d^−1^ capita^−1^) of food *i* by the population in province *j*. BW represents average body weights of Chinese adult males and females, which are reported as 66.2 kg and 57.3 kg (see Supplementary Table 4), respectively. *C*_*ik*_ is the MeHg concentration in food *i* harvested in province *k*, and EDI_*j*_ represents the EDI of MeHg by the population in province *j*.

### Human health impacts

Neurodevelopmental health endpoint has aroused global concern for maternal bodies with prenatal exposure to low levels of MeHg, which would result in IQ decrements in foetuses^3,26^. The IQ decrements in foetuses result in a delayed neurodevelopment when they are born, and even the neurocognitive impact persists into adulthood^62^. Compared to neurodevelopmental health, substantial uncertainties are observed in the cardiovascular effects of MeHg due to limited epidemiological studies. The association of MeHg exposure and cardiovascular disease has been proposed over 10 years while inconsistent results are still reported^63^. Some scholars suggested a dose–response between MeHg exposure and cardiovascular disease, while others found no association^5,64,65^. For instance, recently, Downer et al.^66^ found no evidence that Hg exposure from regular fish consumption increases cardiovascular disease risk in a large clinical trial in Spain. However, based on the International Polar Year Inuit Health Survey (IHS), Hu et al.^67^ found that Hg exposure could diminish the cardiovascular protective effect of omega-3 polyunsaturated fatty acids (*n*-3 PUFAs), resulting in the increase of cardiovascular disease risk. According to the highlighted potential for MeHg exposure to reduce the beneficial effect of *n*-3 PUFAs in previous studies, we tend to suggest that dietary MeHg exposure is a synergistic factor in the acceleration of the occurrence of fatal heart attacks but not a direct cause of the death from fatal heart attacks, which is referred to as Hg-related fatal heart attacks in this study. Meanwhile, in terms of the recommendation by a recent workshop from the United States Environmental Protection Agency (EPA) which suggests sufficient evidence for the dose–response relationship^5,26^, and the studies by Rice et al.^64^ and Giang and Selin^26^, we include fatal heart attacks as a critical Hg-related health impact in this study. It is worth noting that these adverse health impacts are also influenced by many other intrinsic factors which are not considered in this study (see Supplementary Fig. 8).

The dose–response relationships between dietary intake of MeHg and Hg-related health impacts and associated coefficients were suggested by previous studies^5,26,64^ which were based on epidemiological studies^68–70^. Meanwhile, the other input data regarding the number of people and the incidence of fatal heart attacks are taken from the official statistics in China. In addition to dietary MeHg intake, the current incidence of fatal heart attacks among people is another important factor potentially affecting Hg-related fatal heart attacks. Healthy dietary habits and improved medical treatment might increase the protective effects of *n*-3 PUFAs, subsequently decrease the incidence of fatal heart attacks, and eventually decrease the deaths from Hg-related fatal heart attacks. The goal of this study is to distinguish the multiple-perspective emission sources of Hg-related health risks in China and evaluate the impacts of interprovincial trade. Due to this goal, we only consider the factor of dietary MeHg intake driven by anthropogenic Hg emission sources in this study.

First, we evaluate the IQ effects. Based on the review of previous epidemiologic studies^68–70^, the National Research Council (NRC) has recommended the use of a linear dose–response relationship between maternal intake of MeHg and foetal IQ decrements in the absence of significant evidence for another form^4^. The associated coefficients for the relationship were characterized by these epidemiologic studies simultaneously. Previous studies, such as those of Rice et al.^64^ and Giang and Selin^26^, have improved the relationship and applied it into practical cases in the United States. Following these studies, we apply the linear dose–response relationship without thresholds between maternal intake of MeHg and foetal IQ decrements to assess the IQ effects caused by MeHg in China. The assessment of the IQ effects is shown below:

11 $${\mathrm{\Delta IQ}} = \gamma \lambda \beta ({\mathrm{\Delta EDI}} \cdot {\mathrm{BW}}),$$

where ΔIQ represents the changes in IQ points; ΔEDI indicates the changes in EDI of MeHg; and BW represents the average body weight for Chinese female adults. The coefficients *β* (μg Hg L^−1^ blood per μg Hg day^−1^), *λ* (μg Hg g^−1^ hair per μg Hg L^−1^ blood), and *γ* (IQ points per μg Hg g^−1^ hair) represent the conversion factors of MeHg from intake to blood, blood to hair, and hair to IQ, respectively. The values for the coefficients are referred to the study of Rice et al.^64^ which is based on previous epidemiologic studies (see Supplementary Table 4).

Second, we evaluate the cardiovascular impacts. An epidemiological study called the European Community Multicenter Study of Antioxidants, Myocardial Infarction and Breast Cancer (EURAMIC) suggested that the shape of the dose–response function for Hg-related fatal heart attacks was log-linear relationship^65^. The epidemiologic study characterized the associated coefficients for the relationship simultaneously. Previous studies, such as those of Rice et al.^64^ and Giang and Selin^26^, have also improved and applied the relationship. We take the dose–response relationship as the following form according to the method of Rice et al.^64^:

12 $${\mathrm{\Delta CF}} = \mathop {\sum}\limits_{g} {{\kern 1pt} {P}_{g} \cdot {\mathrm{Cf}}_{g} \cdot \omega \cdot (1 - {\mathrm{exp}}( - \varphi \lambda \beta ({\mathrm{\Delta EDI}} \cdot {\mathrm{BW}})))},$$

where ΔCF represents the changes in the deaths from fatal heart attacks due to changes in the intake of MeHg, *P*_*g*_ is the number of people aged ≥40 years of gender *g*, and Cf_*g*_ indicates the current incidence of fatal heart attacks among people aged ≥40 years of gender *g*. The coefficient *φ* (risk per μg Hg g^−1^ hair) represents the conversion factors of MeHg in the hair biomarker to fatal heart attack risks. In terms of the uncertainties for the causality of the association between hair MeHg level and heart attack risks, the coefficient *ω* is introduced to represent the probability of the causality of the associations, which is assigned as a subjective probability of one-third for the causal epidemiological associations (i.e., *ω* = 1) and two-thirds for no causal associations (i.e., *ω* = 0)^64^. BW represents the average body weights for Chinese male and female adults. We also include the coefficient of heart attack cessation lag (τ) to estimate the length of time between changed MeHg intake and the response of fatal heart attacks. The central tendency is estimated as 6 years. Thus, the *P*_*g*_ and Cf_*g*_ data are collected in the year 2015 to represent the impacts of MeHg intake in 2010. The *P*_*g*_ and Cf_*g*_ data are provided in Supplementary Data 1 and Table 5, respectively, and the values for the coefficients are provided in Supplementary Table 4.

## Supplementary information

Supplementary Information

Description of Additional Supplementary Files

Supplementary Data 1

Supplementary Data 2

Supplementary Data 3

Supplementary Data 4

Supplementary Data 5

Supplementary Data 6

Supplementary Data 7

Supplementary Data 8

Supplementary Data 9

Supplementary Data 10

Source Data

## Data Availability

The data regarding the MRIO analyses include the production-based emission inventory and China’s MRIO table, which can be obtained from previous literature^35,42^. The data regarding the simulation of chemical transport model (GEOS-Chem) can be obtained from the model’s website (http://geos-chem.org). The geographic information layers for China’s administrative regions used in the graphs of this study were obtained from the National Geomatics Center of China (http://www.webmap.cn). The data regarding the compilation of intake inventory of MeHg include the concentrations of THg and MeHg in food products provided in Supplementary Data 4, output values of food products provided in Supplementary Data 6, yields of fish in Chinese coastal provinces provided in Supplementary Table 3, and intake rates of food products provided in Supplementary Data 7 and 8. The data regarding the evaluation of human health impacts include the coefficients for dose–response relationships provided in Supplementary Table 4, number of people and incidence of fatal heart attacks provided in Supplementary Data 1 and Table 5. The source data underlying Figs. 1–4 and Supplementary Figs. 1, 2, and 5–7 are provided as a Source Data file. All datasets generated during this study are available from the corresponding authors upon reasonable request.

## Code availability

All computer codes generated during this study are available from the corresponding authors upon reasonable request.
