# Supplementary material for: Trans-provincial health impacts of atmospheric mercury emissions in China
Source: Nat Commun. 2019 Apr 2;10:1484. doi: 10.1038/s41467-019-09080-6 (PMC6445112; doi:10.1038/s41467-019-09080-6)
Supplement: Supplementary file 1 — Supplementary Information [file 41467_2019_9080_MOESM1_ESM.pdf]

Supplementary Information

Trans-provincial health impacts of atmospheric mercury emissions in China

Chen et al.

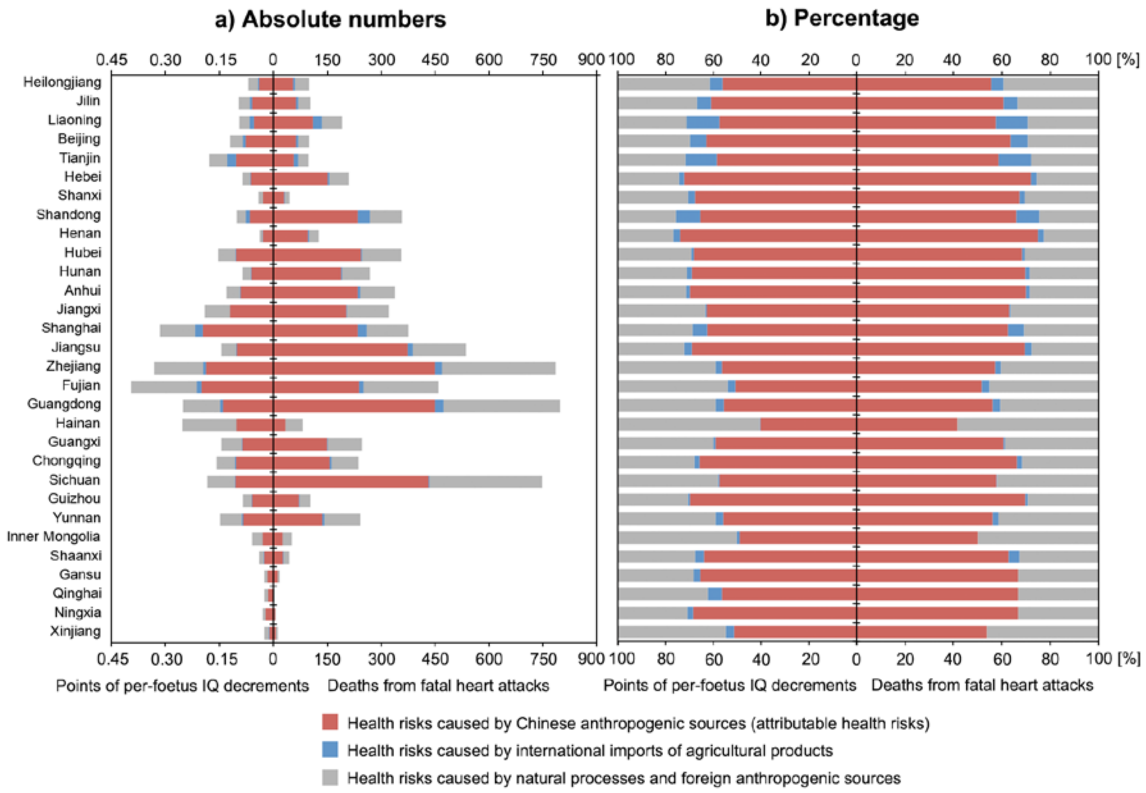

Supplementary Figure 1. Contributions of anthropogenic and natural Hg emission sources to points of per-foetus intelligence quotient (IQ) decrements and deaths from fatal heart attacks in China, including Chinese anthropogenic sources, international imports of agricultural products, and natural sources and foreign anthropogenic sources. Panel a shows the absolute numbers of the points and deaths, and panel b shows the percentage of the points and deaths.

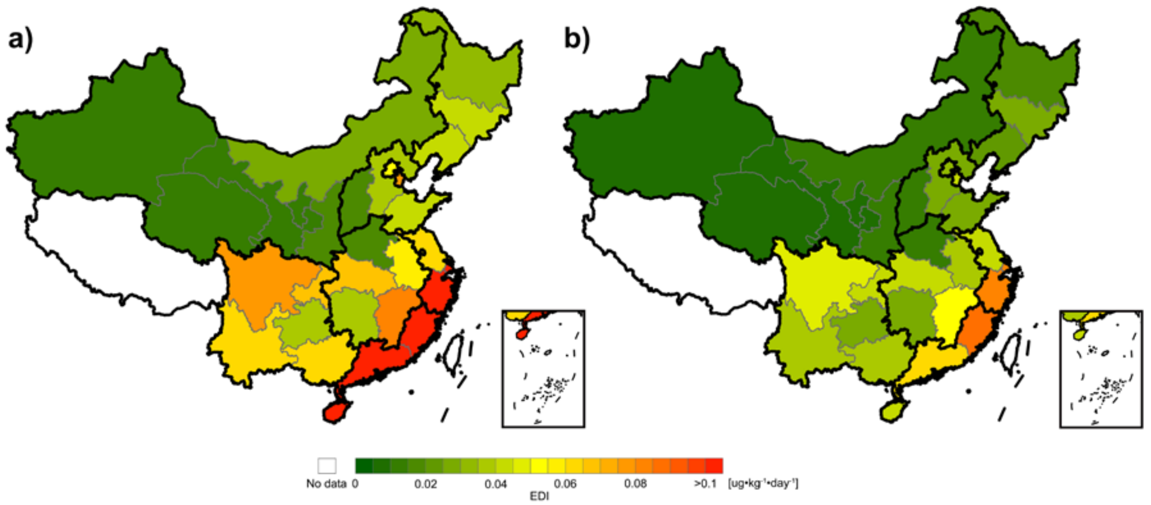

Supplementary Figure 2. Spatial distributions of estimated daily intake (EDI, μg·kg^{-1}·day^{-1}) for the population in China. Panel a illustrates the total EDI caused by all Hg emission sources, and panel b illustrates the EDI caused by Chinese anthropogenic Hg emissions.

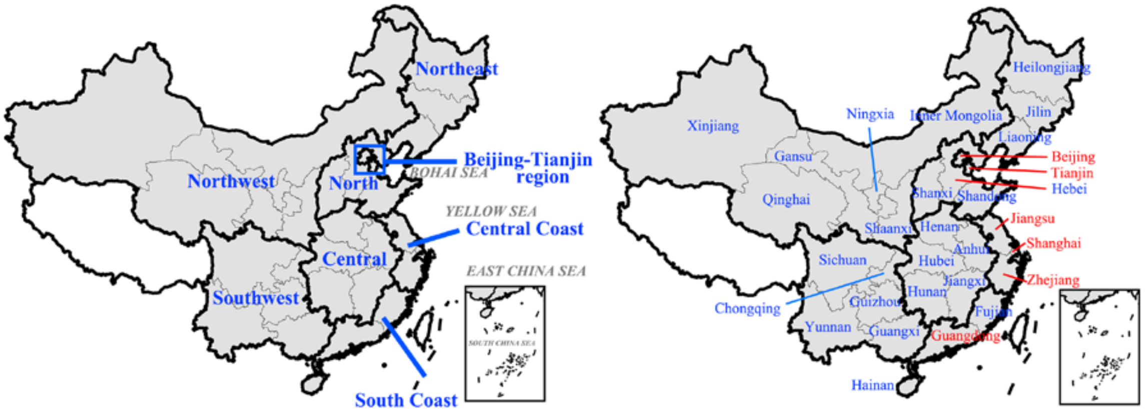

Supplementary Figure 3. Geographical boundaries for regions, provinces, and seas in China. Beijing, Tianjin, Shanghai, Jiangsu, Zhejiang, and Guangdong are developed provinces which are marked in red, and the rest are developing provinces. Tibet, Taiwan, Hong Kong, and Macau are not included in this study due to data unavailability.

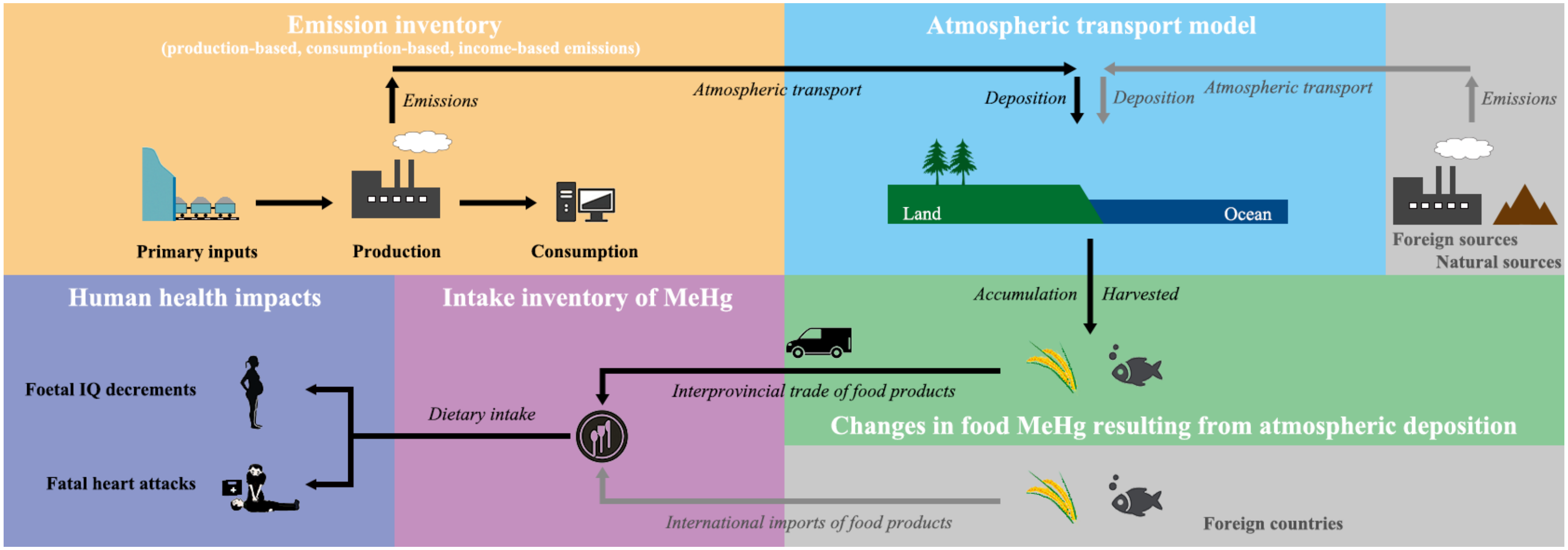

Supplementary Figure 4. Framework of the China Mercury Risk Source-Tracking Model (CMSTM) in this study. The processes located in orange, blue, green, purple, and blue-purple rectangles occur over mainland China and coastal seas, while the processes located in gray rectangle occur over foreign countries or represent natural processes.

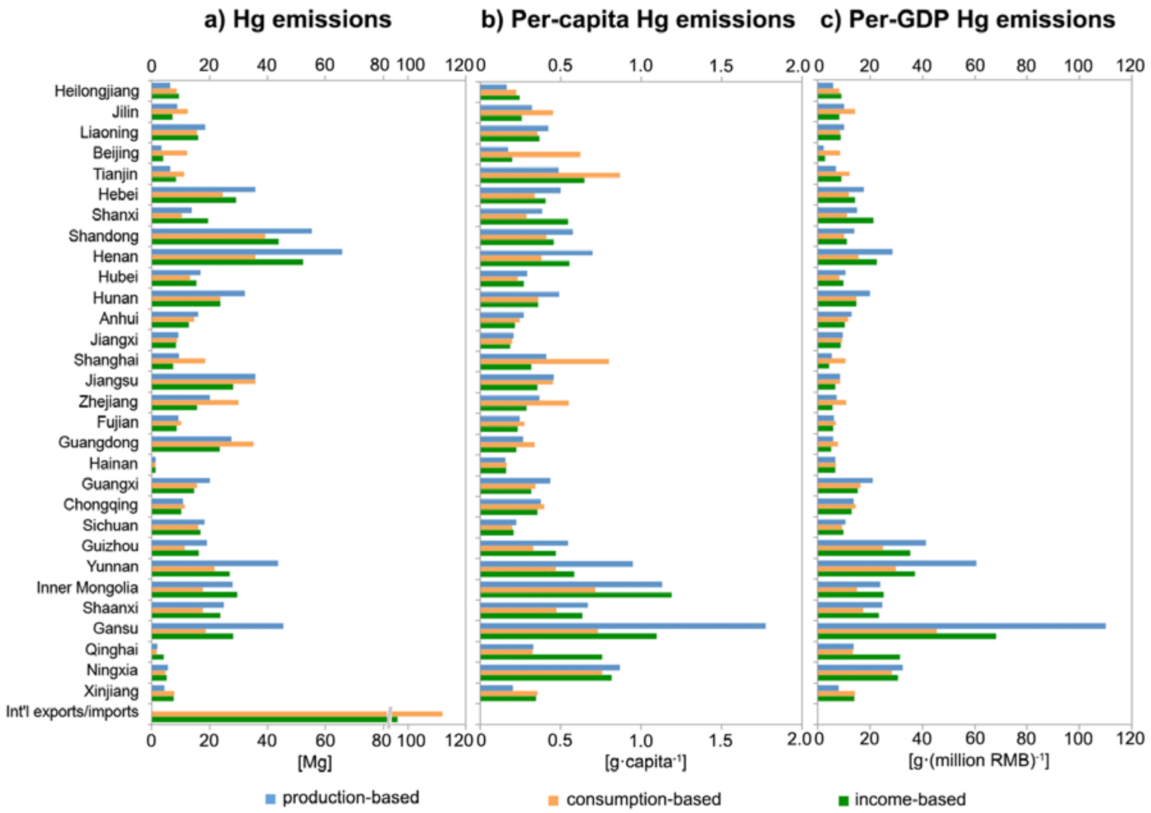

Supplementary Figure 5. Atmospheric Hg emissions from geographical sources over China in 2010. Panel a illustrates total Hg emissions from each geographical source, while panel b and c illustrate per-capita Hg emissions and per-GDP Hg emissions from each geographical source, respectively.

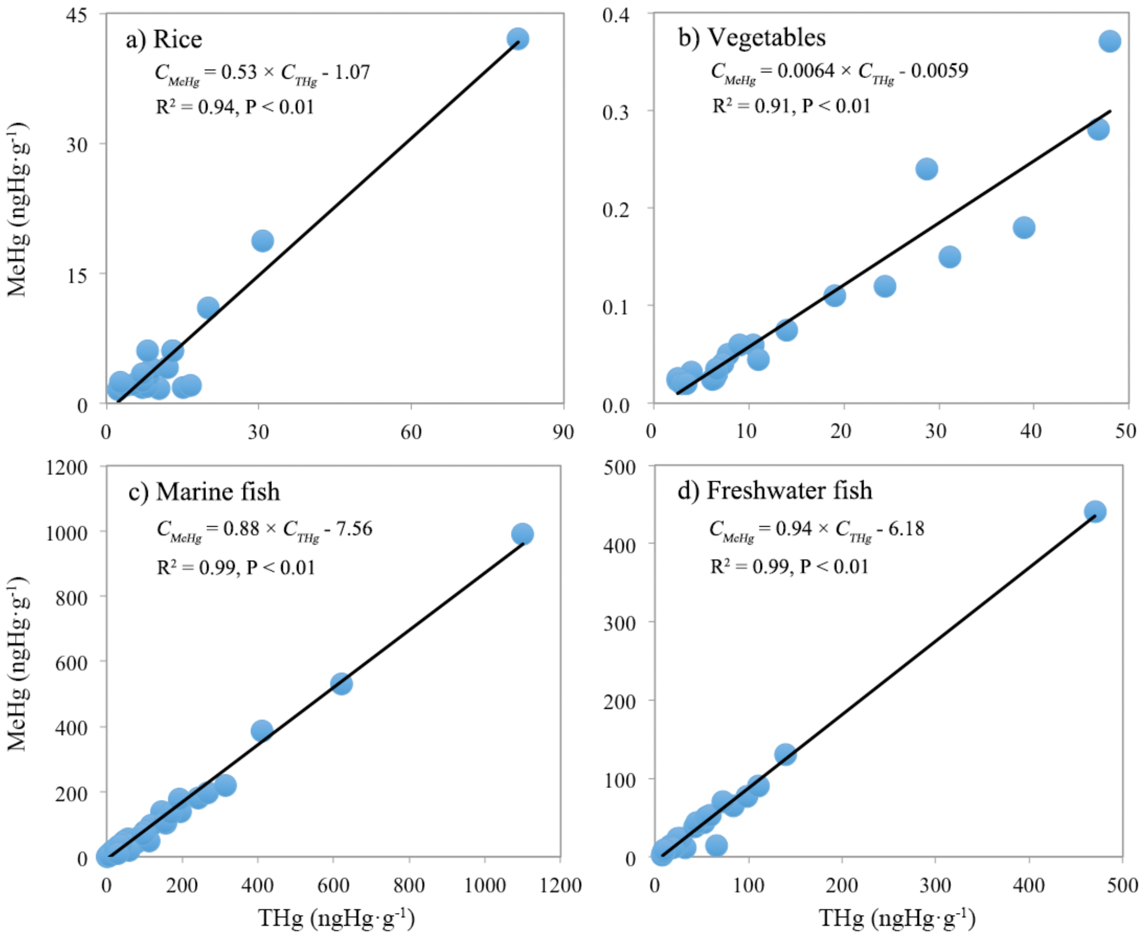

Supplementary Figure 6. Relationships between MeHg concentrations (C_{MeHg}, ngHg·g^{-1}) and THg concentrations (C_{THg}, ngHg·g^{-1}) in rice, vegetables, marine fish, and freshwater fish.

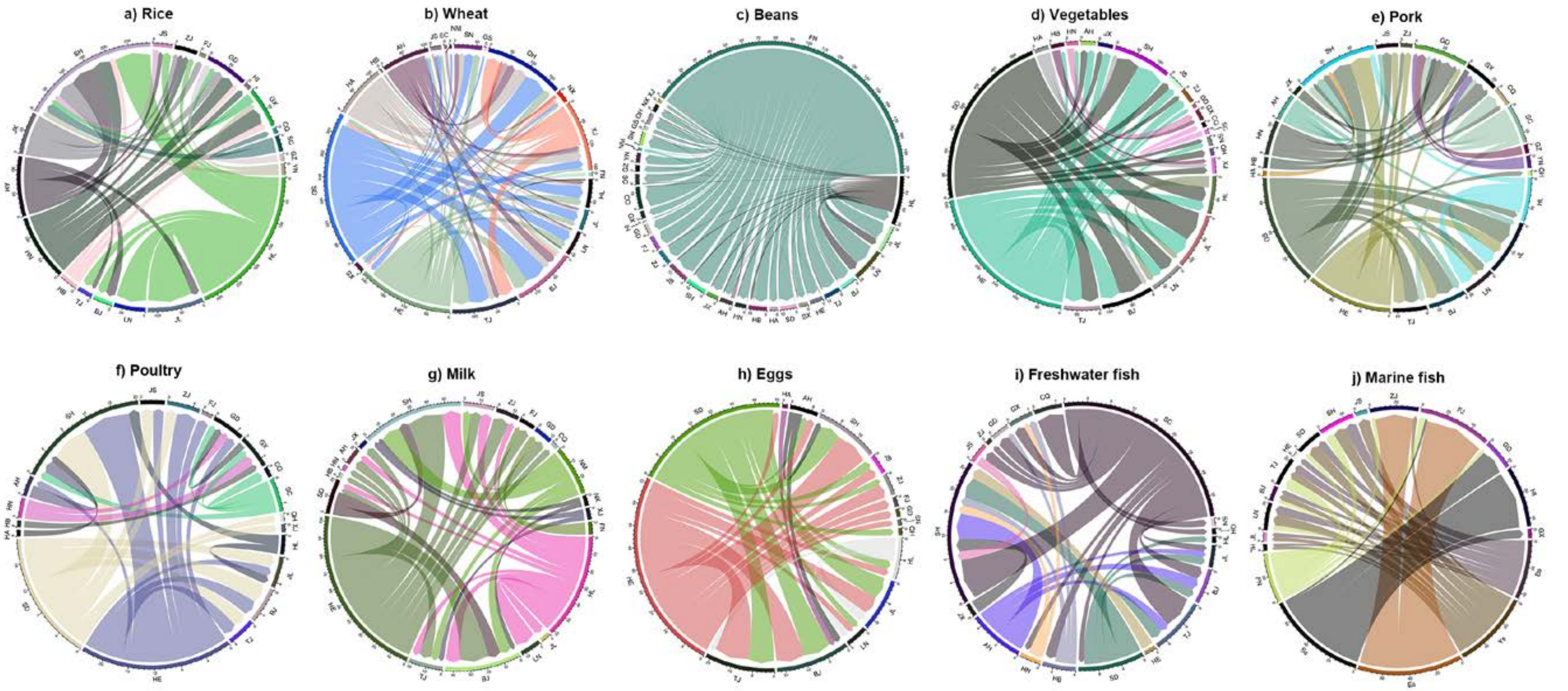

Supplementary Figure 7. Simulated trade of food products among Chinese provinces (g·d^{-1}·capita^{-1}). The abbreviations are HL, Heilongjiang; JL, Jilin; LN, Liaoning; BJ, Beijing; TJ, Tianjin; HE, Hebei; SX, Shanxi; SD, Shandong; HA, Henan; HB, Hubei; HN, Hunan; AH, Anhui; JX, Jiangxi; SH, Shanghai; JS, Jiangsu; ZJ, Zhejiang; FJ, Fujian; GD, Guangdong; HI, Hainan; GX, Guangxi; CQ, Chongqing; SC, Sichuan; GZ, Guizhou; YN, Yunnan; NM, Inner Mongolia; SN, Shaanxi; GS, Gansu; QH, Qinghai; NX, Ningxia; XJ, Xinjiang; FN, foreign countries (food products imported from foreign countries); Bs, Bohai Sea; Ys, Yellow Sea; Es, East China Sea; Ss, South China Sea. The panels show major flows for each type of food products, with the flow quantities larger than 10, 5, 0.1, 15, 3, 1, 1, 3, 2, 1, and 2 g·d^{-1}·capita^{-1} for rice, wheat, beans, vegetables, pork, poultry, milk, eggs, freshwater fish, and marine fish, respectively.

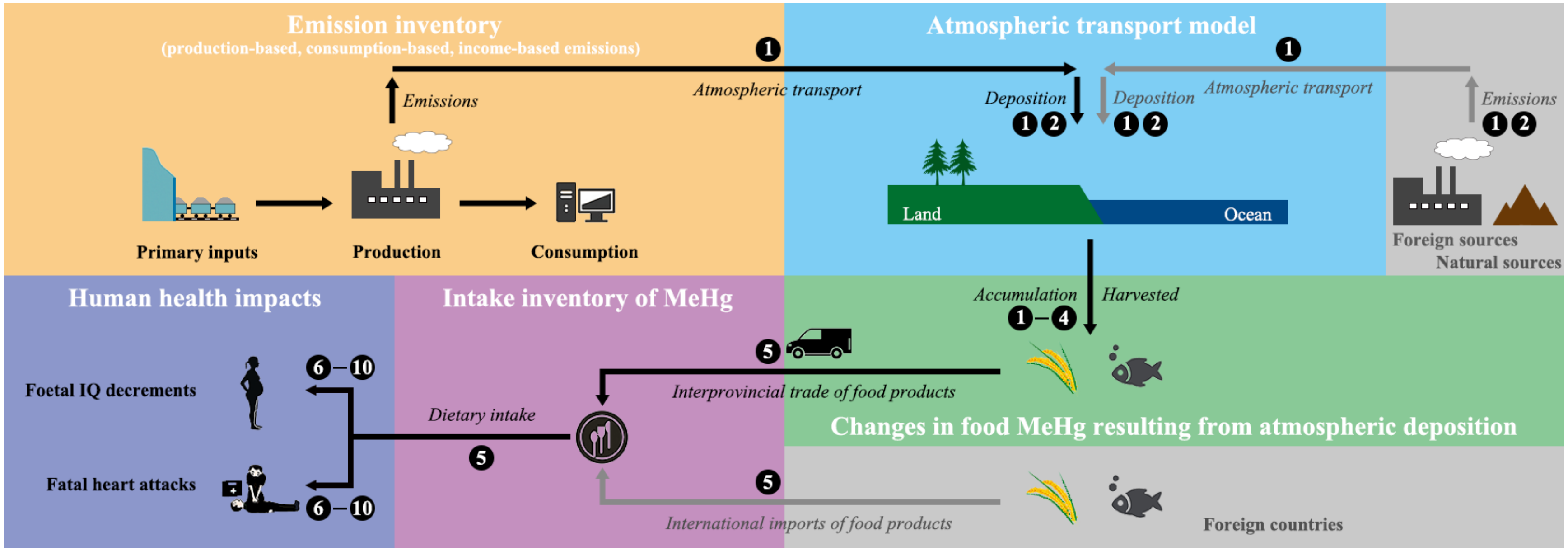

Supplementary Figure 8. Processes of the China Mercury Risk Source-Tracking Model (CMSTM) potentially influenced by extrinsic and intrinsic factors. The extrinsic factors include climate change (1), land use change (2), hydrologic management (3), invasive species (4), and food consumption & dietary habits (5). The intrinsic factors include genetics (6), gastrointestinal assimilation (7), microbiome (8), nutrients & co-exposures to other contaminants (9), and co-exposures to other diseases (10).

Supplementary Table 1. Sector classification of the Chinese multiregional input-output (MRIO) table

Sector index	Sector names	Shorthand versions
S1	Farming, Forestry, Animal Husbandry and Fishery	Agriculture
S2	Coal Mining and Dressing	Coal Mining
S3	Petroleum and Natural Gas Extraction	Petroleum and Gas Extraction
S4	Metals Mining and Dressing	Metals Mining
S5	Nonmetal and Other Minerals Mining and Dressing	Other Minerals Mining
S6	Food Production and Tobacco Processing	Food Production
S7	Textile Industry	Textile Industry
S8	Garments, Leather, Furs, Down and Related Products	Leather
S9	Timber Processing and Furniture Manufacturing	Timber Processing
S10	Papermaking, Printing, Cultural, Educational and Sports Articles	Paper and Printing
S11	Petroleum Processing and Coking	Coking
S12	Chemical Industry	Chemical Industry
S13	Nonmetal Mineral Products	Nonmetal Mineral Products
S14	Smelting and Pressing of Metals	Metals Smelting
S15	Metal Products	Metal Products
S16	General and Special Equipment	General Equipment
S17	Transportation Equipment	Transportation Equipment
S18	Electric Equipment and Machinery	Electric Equipment
S19	Electronic and Telecommunications Equipment	Electronic Equipment
S20	Instruments, Meters Cultural and Office Machinery	Office Machinery
S21	Other Manufacturing Industry	Other Manufacturing
S22	Production and Supply of Electricity and Heat Power	Electricity and Heat Power
S23	Production and Supply of Gas and Water	Gas and Water
S24	Construction	Construction
S25	Transport and Storage	Transport and Storage
S26	Wholesale and Retail	Wholesale and Retail
S27	Hotels and Catering Services	Hotels and Catering
S28	Leasehold and Business Services	Leasehold and Business
S29	Scientific Research	Scientific Research
S30	Other Services	Other Services

Supplementary Table 2. Correspondence relationships between emission sources and sectors of the Chinese MRIO table

Emission sources		Corresponding sector in the Chinese MRIO table
**Large category**	**Sub-category**	
Fuel consumption	Coal-fired power plants	Production and Supply of Electricity and Heat Power
	Industrial coal combustion	Disaggregated to 30 sectors
	Residential coal combustion	Not included
	Other coal combustion	Disaggregated to 30 sectors
	Stationary oil combustion	Disaggregated to 30 sectors
	Mobile oil combustion	Disaggregated to 30 sectors
Incineration and cremation	Municipal solid wastes incineration	Other Services
	Biomass combustion	Farming, Forestry, Animal Husbandry and Fishery
	Cremation	Other Services
Nonferrous metal smelting	Copper smelting[a]	Smelting and Pressing of Metals
	Lead smelting[a]	Smelting and Pressing of Metals
	Zinc smelting[a]	Smelting and Pressing of Metals
	Large-scale gold production	Smelting and Pressing of Metals
	Artisanal and small-scale gold mining	Smelting and Pressing of Metals
	Aluminum production	Smelting and Pressing of Metals
Primary mercury mining	Primary mercury mining	Metals Mining and Dressing
Building materials production	Cement production[b]	Nonmetal Mineral Products
	Iron and steel smelting	Smelting and Pressing of Metals
Intentional use	Chlor-alkali production	Chemical Industry
	Thermometer production	Instruments, Meters Cultural and Office Machinery
	Sphygmomanometer production	Instruments, Meters Cultural and Office Machinery
	Fluorescent lamp production	Electric Equipment and Machinery
	Battery production	Electric Equipment and Machinery
	Use of Hg-added products	Not included
Hg recovery	Hg production from recyclable resources	Smelting and Pressing of Metals

Notes: (a) Including secondary emissions from waste disposal (i.e., slags) and recovery of oxidized metals in smelters; (b) Including secondary emissions from waste disposal in cement plants and the utilization of fly ash for bricks production and gypsum for wallboard production.

Supplementary Table 3. Yields of marine fish and freshwater fish in Chinese coastal provinces (t)[a]

Provinces	Mariculture				Marine fishing					Total
	Bohai Sea	Yellow Sea	East China Sea	South China Sea	Bohai Sea	Yellow Sea	East China Sea	South China Sea	Pelagic fishery	
Beijing									6278	6278
Tianjin	14212				8467	7287			4088	34054
Hebei	329308				192577	60715				582600
Liaoning	1157347	1157347			391061	576124	40213		103803	3425895
Shanghai							21531		26338	47869
Jiangsu		785173			275	504763	65316		4848	1360375
Zhejiang			825730			203690	2597690	19620	141054	3787784
Fujian			3038990				1716721	191747	133645	5081103
Shandong	1981322	1981322			476318	1695807	177463	1300	58551	6372083
Guangdong				2490688				1429592	38852	3959132
Guangxi				877408				662954		1540362
Hainan				184162				994715		1178877
Total	3482189	3923842	3864720	3552258	1068698	3048386	4618934	3299928	517457	

Notes: (a) Data are taken from China Fisheries Yearbook 2011[1].

Supplementary Table 4. Parameters used in the evaluation of health impacts from MeHg intake

Symbol	Definition	Unit	Category	Distribution Type	Central Tendency	Variability	Reference
BW	Body weight	kg	Male<br>Female		66.2<br>57.3		Chang & Wang[2]
P_g	Number of people ≥40, gender g	Per year			Supplementary Data 1		
Cf_g	Number of heart attacks of age g	Per year			Supplementary Table 5		
γ	IQ-hair mercury coefficient	µg Hg/g hair		Uniform	0.3	[0.0135, 0.567]	Axelrad et al.[3]; Rice et al.[4]; Giang & Selin[5]
λ	Hair-blood coefficient	µg Hg/g hair per µg Hg/L blood	Pregnant women	Normal	0.2	0.0014	Shipp et al.[6]; Allen et al.[7]
			Nonpregnant women and other adults	Normal	0.18	0.0014	
β	Blood-intake coefficient	µg Hg/L blood per µg Hg/day		Normal	0.6	0.09	Stern[8]
φ	Heart attack-hair mercury coefficient	Risk per µg Hg/g hair		Triangular	0.066	[0, 0.17]	Salonen et al.[9]
τ	Heart attack cessation lag	Year		Uniform	6	[2, 12]	Rosenberg et al.[10]; Willett et al.[11]; Dobson et al.[12]; Bakhru & Erlinger[13]
ω	Plausibility of causality-heart attacks vs mercury	Dimensionless	1/3 MeHg causally associated with heart attack		1		Rice et al.[4]
			2/3 epidemiologic associations not causal		0		

Supplementary Table 5. Incidence of fatal heart attacks among Chinese populations in 2015 by domiciles, ages, and genders (per-hundred-thousand people)[a]

Domicile	Gender	Age									
		40-44	45-49	50-54	55-59	60-64	65-69	70-74	75-79	80-84	≥85
Urban	Male	16.08	21.92	55.13	53.83	107.94	176.55	262.22	455.67	877.06	2059.55
	Female	4.55	6.48	15.43	18.29	46.02	95.72	180.91	351.83	761.01	2001.96
Rural	Male	20.89	32.24	62.70	70.10	126.02	219.32	343.70	588.94	1066.30	2458.43
	Female	6.22	9.87	23.70	28.58	68.68	146.55	266.00	449.70	883.40	2317.87

Notes: (a) The incidence data are taken from China Health and Family Planning Statistical Yearbook (2016)[14].

Supplementary Discussion

Comparisons with other studies

A series of articles have reported anthropogenic Hg emissions and atmospheric Hg deposition embodied in the final consumption and interprovincial trade in China[15-18], revealing the importance of the virtual transboundary flows in Hg issues in China. Compared to these studies that investigated a shorter chain of the biogeochemical Hg cycle from economic activities to atmospheric Hg emissions and deposition, the investigation of a longer chain of the biogeochemical Hg cycle from economic activities to human health in this study more fully meets the requirements of Hg-related research and policies. Three main improvements are proposed in the discussion section of the main text, and the last one is illustrated here in detail. Additional biological and physical processes are considered in the longer chain of the biogeochemical Hg cycle, including changes in food MeHg resulting from atmospheric deposition, intake of MeHg to human beings, and human health impacts due to MeHg intake. These additional processes provide different results of source identification and relevant policy implications from those provided in previous studies. Supplementary Data 5 shows the contributions of geographical sources to national anthropogenic Hg emissions and atmospheric Hg deposition illustrated in previous studies, and to national Hg-related health risks in this study. Different results are obtained with respect to the different endpoints. For example, compared to the endpoints of atmospheric Hg emissions and deposition, the contributions of Jiangsu and Sichuan to national Hg-related health risks increased, while the contribution of final consumption in Henan to national Hg-related health risks decreased.

Different rankings of geographic sources are observed for different endpoints, which is meaningful for policy decisions. However, the ultimate goal of Hg reductions is to reduce Hg-related health risks to human beings. Thus, the endpoints of atmospheric Hg emissions and deposition reported in previous studies cannot fully support this ultimate goal. The endpoint of human health in this study contributes to supporting more targeted and effective control measures than those applied in previous studies. Moreover, source identification for primary suppliers in this study, which can support supply-side policy decisions to effectively reduce Hg-related health risks, has not been investigated in previous studies.

Uncertainty analysis

Our results are subject to certain uncertainties, and we discuss the uncertainty ranges in different steps of our analysis, including the compilation of Hg emission inventory, the MRIO analyses, the simulation of chemical transport model, the compilation of intake inventory of MeHg, and the evaluation of human health impacts. Finally, the overall uncertainties of production-based, consumption-based, and income-based Hg-related health risks are derived from aggregations of the uncertainties above using Monte Carlo simulations at 10,000 samplings (see Supplementary Data 10 and 11). We obtain the results in the form of statistical distributions and set P10 and P90 values of the distribution curves as the lower and upper limits of the uncertainty range.

(1) The compilation of production-based Hg emission inventory is uncertain due to knowledge gaps regarding Hg concentrations in fuel/raw materials, technology distributions, and activity rates. Since we use a Chinese production-based Hg emission

inventory derived from our previous work[17], we introduce an overall uncertainty of [–25%, 29%] for the production-based emissions from that work.

(2) Estimates of consumption-based and income-based Hg emissions share the most uncertainties with production-based Hg emissions and contain an additional uncertainty from the MRIO model associated with inaccuracies in economic statistics, sectoral mapping, and data harmonization[19-21]. A comparable variability between production-based and consumption-based CO_2 emissions across studies using different MRIO models has been observed in Peters et al.[22], and their study indicated a very small MRIO-related uncertainty compared to the uncertainty in production-based emissions. A comparison of different global MRIO models by Peters et al.[22] suggested a 13% uncertainty for the past estimates of global CO_2 emissions embodied in trade. In the present study, we add a 13% uncertainty to production-based Hg emissions to represent the uncertainty of consumption-based and income-based Hg emissions derived from the MRIO data, consistent with previous studies such as those by Lin et al.[8] and Zhang et al.[23]

(3) Atmospheric Hg deposition simulated by the chemical transport model (GEOS-Chem) is affected by errors in emission inputs and the model representation of tropospheric chemical processes, especially Hg chemistry and physical processes such as vertical transport and wet scavenging. It is infeasible to estimate related uncertainties via Monte Carlo simulations or sensitivity analysis that require a large number of tests due to the large computational intensity of the chemical transport model[24,25]. Based on the method described by Zhang et al.[23], we instead use the normalized root-mean-square deviation (NRMSD) between the simulated and observed atmospheric Hg deposition over

measurement sites to represent the uncertainties derived from the chemical transport model. The NRMSD is estimated as 23.12% in this study.

(4) The compilation of intake inventory of MeHg is subject to uncertainties in the MeHg concentrations of food products, trade activities of food products, and intake rate of food products. MeHg concentrations of food products are collected from the literature. Measurements in each region have multiple data for estimating the standard deviation (see Supplementary Data 4). Similar to consumption-based and income-based Hg emissions, the simulation of trade activities of food products is also subject to the uncertainty from the MRIO data. Thus, we also add a 13% uncertainty to the simulation. Since uncertainties are not provided for the yearbook statistics of per-capita consumption, we assume a 10% uncertainty for the national yearbook statistics according to the UNEP report[26] and Wu et al.[27] However, the population variability in intake is substantial. For example, the populations living in coastal areas in Fujian, Zhejiang, and Guangdong experience high dietary intake of marine fish. An assumed 10% uncertainty might not fully represent the substantial variability in the intake in some specific provinces, but data are unavailable for sub-populations in a given province. Future studies are expected to investigate the dietary intake of certain food products for sensitive sub-populations. Monte Carlo simulations are used to produce the probabilistic intake of MeHg by considering the probability distribution of the key parameters above for each region.

(5) The evaluation of human health impacts due to MeHg intake is subject to uncertainties in parameters used in the evaluation and the total deaths from fatal heart attacks collected from national statistics. The parameters are referred to in previous studies[3-5], and each of them is in accordance with a probability distribution function (see

Supplementary Table 4 and Data 10). We use the probability distribution functions in the Monte Carlo simulations to represent the uncertainties from the parameters. The total deaths of fatal heart attacks are calculated by multiplying the number of people aged ≥ 40 years with the current incidence of fatal heart attacks among people aged ≥ 40 years, which are collected from Sample Survey Data of 1% of National Population[28] and China Health and Family Planning Statistical Yearbook[14], respectively. Due to the lack of uncertainties reported in these national statistics, we assume a 10% uncertainty for these data following the common practice used by Lin et al.[25], Wu et al.[27], and the UNEP report[26].

Limitations and prospects

In addition to the uncertainties discussed above, there are additional limitations in this study. For instance, we assume that the food products whose THg or MeHg concentrations exceed the contaminant limits of the Chinese government[29] would not be allowed to circulate in markets, and they are hence not considered in our analysis. That is, in this study, we consider food products from background soils and waters influenced by atmospheric Hg deposition and do not consider food products from local contamination areas with polluted soils and waters. These patterns of Hg pollution primarily include soil contamination in mining areas and sewage irrigation and aquaculture in some critical provinces. However, the “old” Hg adsorbed in soil has a lower affinity to undergo methylation than newly atmospheric deposited Hg, and atmospheric deposited Hg is more bioavailable for microbial methylation than Hg forms with an extended residence time in soil[30,31]. Therefore, we speculate that atmospheric deposition is still the primary source of

MeHg in food products from soil contamination sites in mining areas. The Chinese government has controlled the sewage irrigation and aquaculture since the 21th century and prohibited these activities until 2013[32]. Although direct Hg discharges to soils and waters have been reported by previous studies, which is approximately 50 Mg yr^{-1} in Liu et al.[33] (equivalent to 8% of atmospheric Hg emissions), the actual Hg pollution on areas for planting and aquaculture was limited. If we consider these patterns of Hg pollution, the attributable health risks induced by Chinese anthropogenic Hg emissions would be smaller in some critical provinces (e.g., Tianjin, Beijing, and Guizhou). However, due to the limited impacts, the reduced proportion would be limited. Meanwhile, because most results of our study are presented in the form of ratios and differences, they would not be changed when considering the pollution of local Hg contamination. However, these patterns of Hg pollution are more important for regional-scale (e.g., city) studies in the future.

Previous studies have shown that ecosystems can respond to changes in Hg inputs on timescales of years to decades[34,35]. Hg concentrations in food products collected from the literature in this study were measured during recent decades and are used to represent the average condition of Hg risks in these decades. Hg emissions and atmospheric Hg transport in 2010 can represent an average condition of Hg emissions during the last few decades. We establish relationships between Hg emissions and risks during the last few decades and do not consider the accurate lag time in the response between Hg emissions and risks for a specific time period. Additional measurements of yearly data on Hg concentrations in food products and accurate lag time in the response between Hg emissions and risks are needed in the future.

The China Mercury Risk Source-Tracking Model (CMSTM) in this study focuses on the impacts of human activities in the economic supply chains to Hg-related health risks. In addition to economic supply chains and Hg emission sources, the biogeochemical Hg cycle and related adverse health impacts (especially the human exposures) are also influenced by multiple extrinsic and intrinsic factors. The extrinsic factors include climate change, land use change, hydrologic management, invasive species, and food consumption & dietary habits. The intrinsic factors include genetics, gastrointestinal assimilation, microbiome, nutrients & co-exposures to other contaminants, and co-exposures to other diseases.

The CMSTM in this study has not considered these factors mainly due to two reasons: (1) The primary aim of this study is integrating the economic supply chains with Hg-related health impacts to identify diverse sources of the adverse health impacts in large human communities. Thus, we conduct a series of simulations at various source scenarios for a single year to achieve this aim. The values of parameters describing certain extrinsic factors are determined for the single year according to model simulations and national statistics, such as the air temperature, precipitation, land use types, leaf area index, and per-capita food consumption. The CMSTM can be extended to a temporal dynamic model when involving the dynamic changes of these parameters. Such a dynamic model can track the changes in Hg cycle and related health risks in the context of rapid global changes. However, this point is beyond our research goal of this study. (2) Intrinsic factors are important for the assessment of Hg-related health risks. However, findings of existing studies for some intrinsic factors (e.g., genetics and co-exposures to other diseases) are inconsistent with one another, and the mechanisms of some intrinsic

factors (e.g., microbiome) still remain unknown. This prevents the adoption of these intrinsic factors in the assessment of Hg-related health risks. Moreover, intrinsic factors are mostly at the individual and molecular levels, which may be inappropriate for studies on large human communities at the macro scale. Findings from epidemiological studies can be applied to studies on large human communities at the macro scale, because their statistical conclusions can simplify the unknown mechanisms during exposure processes and hence improve the feasibility of the studies.

In general, the CMSTM in this study has not considered these extrinsic and intrinsic factors. However, these factors can be practicably incorporated into the CMSTM, when future studies can clearly characterize their dynamics, mechanisms, and modelling methods. In the following paragraphs, we will discuss potential impacts of these extrinsic and intrinsic factors on the Hg cycle and related health risks and the processes in the CMSTM of this study.

First, we investigate extrinsic factors, including climate change, land use change, hydrologic management, invasive species, and food consumption & dietary habits.

Climate change influences the bioaccumulation and biomagnification of MeHg in food webs through altering the primary productivity, food web structure, and bioenergetics[36]. For example, in order to maintain the higher metabolic rate associated with warmer water temperatures, some organisms would increase the food consumption and subsequently result in a substantial increase of their MeHg concentrations[37]. Thus, climate warming will exacerbate methylation and bioaccumulation of Hg in aquatic ecosystems in the future[36]. In addition to food webs, climate change can significantly influence the Hg chemistry and deposition in the atmosphere. High temperature can

decrease the oxidation of Hg^0 by Br atoms and high moisture can increase aqueous photo-reduction of Hg^{II} [38]. Climate change induces the changes in precipitation patterns, which can change the spatial distribution, magnitude, and seasonal variation of atmospheric Hg wet deposition[38]. Furthermore, climate change can also influence the frequency of biomass burning releasing atmospheric Hg. It is observed that wildfire-related Hg emissions increased by 14% due to climate change[39]. In the polar oceans, rapid changes in sea-ice cover and the seasonality can affect the air–sea exchange of Hg[40]. In terms of these impacts, climate change can probably influence the processes of atmospheric transport, atmospheric deposition, natural emissions, and accumulation of Hg in the CMSTM (Supplementary Figure 8).

Land use change includes the production and discharge of wastes, expansion of cities, conversion of forests to pastures for livestock and crop monocultures (i.e., deforestation), and addition of fertilizers and pesticides to soils. It can disturb natural hydrologic, geochemical, and biological processes of terrestrial ecosystems and subsequently alter the fate of Hg in food webs[41,42]. For example, the expansion of cities and deforestation would induce habitat losses, which can result in losses of sensitive species and biodiversity and subsequently affect food web structure for the bioaccumulation and biomagnification of Hg. In addition to influencing Hg bioaccumulation in food webs, land use change can also affect the fate of Hg in the atmosphere. For instance, increased vegetation and foliage density can increase atmospheric dry deposition and evaporated emissions, while the increases of agricultural land areas would lead to the losses of leafy areas and decrease the dry deposition[36,38]. In terms of these impacts, land use change can probably influence the processes of

atmospheric deposition, natural emissions, and accumulation of Hg in the CMSTM (Supplementary Figure 8).

The hydrology influences the biogeochemical processes of MeHg production, as well as habitat availability and uses, food web structure, and bioenergetics for MeHg[42]. For example, THg and MeHg concentrations would increase by 3-fold to 30-fold within a few years after reservoir creation for aquatic food web components, while the contributions may eventually return to the near pre-impoundment levels after several decades[43,44]. The management on the magnitude of inter-annual changes in maximum water levels is linearly correlated with fish Hg concentrations in subsequent years[44]. Moreover, the hydrologic management of agricultural wetlands such as rice paddies can led to pronounced seasonality in sediment and aqueous MeHg concentrations[45]. The hydrologic management may influence the Hg accumulation process in the CMSTM (Supplementary Figure 8).

The worldwide intentional and unintentional transport of microbes, flora, and fauna occurs due to long distance transportation and globalization[46]. Invasive species have been verified to profoundly affect ecosystem processes and food web structure. However, their impacts on the Hg cycle still remain unclear. Eagles-Smith et al.[42] reported that, through changing the site-specific hydrology, biogeochemistry, and microbial processes, invasive species might influence the movement of MeHg throughout food webs. Invasive species could also change the foraging habitat of organisms which are associated with MeHg bioaccumulation, resulting in the changes of Hg fate in food webs[47]. Moreover, invasive species may serve as new forage species in existing food webs to change the length of trophic pathways, and then affect the Hg cycle within the food webs. Thus, the invasive

species may influence the Hg accumulation process in the CMSTM (Supplementary Figure 8).

The socioeconomic status results in the differences in Hg exposures across communities due to different exposure pathways and dietary habits[48]. For example, urban anglers with lower income in developing nations (e.g., minority and immigrant populations) can experience high Hg exposure risks due to their consumption of more self-caught fishes than store-bought fishes, and they tend to consume predatory species with the highest Hg concentrations[48,49]. However, the higher-income populations prefer to consume high-end economic marine fishes (e.g., tuna and swordfish) and also experience elevated Hg exposure risks[50,51]. Owing to the different dietary habits, Hg exposure risks across communities will change accompanying the socioeconomic development in the context of globalization. Thus, the socioeconomic status factor can influence the processes of interprovincial trade and international imports of food products and dietary intake in the CMSTM (Supplementary Figure 8).

Second, we investigate intrinsic factors, including genetics, gastrointestinal assimilation, microbiome, nutrients & co-exposures to other contaminants, and co-exposures to other diseases.

The genetic factor has been verified to modify the pathway from actual Hg exposure (measured by dietary intake estimates) to realized Hg exposure (measured by biomarkers) [52,53]. For example, Basu et al.[54] not only found the positive correlation between Hg intake and hair Hg levels, but also found that the relationships between Hg intake and biomarker differed across genotypes. Moreover, Hg-related neurodevelopmental and cardiovascular diseases are found to have genetic underpinnings[54]. Existing studies have inconsistent

findings for the genetic factor, which limits the application of the genetic factor to assess Hg exposure risks[54,55].

Existing studies argued that, assuming that 95%-100% of the ingested MeHg is absorbed into systemic circulation through gastrointestinal assimilation in some risk assessment studies might be inappropriate. Bradley et al.[56] found that the bioavailability and assimilation of MeHg are less than 100% (ranging from 2% to 100%) in a variety of vitro organisms and experimental models. Meanwhile, factors such as the seafood types, cooking methods, and the presence of certain nutrients are found to influence the gastrointestinal assimilation of MeHg. Jadán-Piedra et al.[57] revealed that most of the solubilization and bio-accessibility of Hg took place in the presence of acidic pH and pepsin in the gastric step, while the decrease in the bio-accessibility occurred in the presence of bile salts in the intestinal stage. Thus, more efforts are needed in future studies to uncover the mechanisms between ingested MeHg and Hg exposure risks.

Emerging evidence indicates the complex interactions between the microbiome and health impacts of Hg exposures[42]. For instance, the gut microbiome can change Hg speciation or sequester Hg prior to intracellular accumulation, and subsequently alter the toxicity of Hg[58]. Meanwhile, Hg exposures can change the gut microbiome conversely and subsequently alter certain functional groups related to host physiology and health[59]. Although emerging evidence occurs, many mechanisms for these complex interactions are still unknown.

Nutrients and other contaminants, such as selenium (Se), omega-3 polyunsaturated fatty acids (n-3 PUFAs), lead, arsenic, polychlorinated biphenyls (PCBs), and halogenated organic compounds, can have substantial positive or negative influences on

Hg toxicity[60]. Numerous studies have investigated the antagonistic relationships between Hg and Se, but have no consensus. Many mechanisms have been proposed for the ameliorative effects of Se on Hg toxicity, such as the demethylation of MeHg and the pathway of glutathione antioxidant[42,61,62]. The n-3 PUFAs show protective effects for the cardiovascular disease, while MeHg exposure could increase the disease risk through diminishing the cardiovascular protective effect of n-3 PUFAs[63]. Thus, n-3 PUFAs show substantial antagonism of Hg exposures on the cardiovascular outcome. After co-accumulating with Hg, PCBs and organohalogenated compounds might influence the same physiological systems which are impaired by Hg[42]. Existing inconsistent findings require more efforts in future studies to uncover the clear mechanisms between nutrients & other contaminants and Hg toxicity.

Emerging evidence also shows that Hg can affect the immune function[64], the alteration of which can lead to significant impacts on human health. Moreover, different Hg species and exposure magnitudes would induce different types of immunotoxic responses and different mechanisms of immunotoxicity, respectively[65]. Despite the evidence of Hg immunotoxicity, findings of existing studies have many inconsistencies which challenge the estimations of immunological health impacts in rigorous Hg risk assessments[42].

In terms of the impacts discussed above, all the intrinsic factors can influence the processes of Hg exposures and related adverse health impacts in the CMSTM (Supplementary Figure 8).

Supplementary References

1. Fisheries and Fisheries Administration Bureau of the Ministry of Agriculture. China Fisheries Yearbook, 2011. China Agricultural Press, Beijing, China (2011).

2. Chang, J. L. & Wang, Y. Comprehensive report on the monitoring of Chinese residents' nutrition and health status during 2010 to 2013. Medical Press of Peking University, Beijing, China (2016).

3. Axelrad, D. A., Bellinger, D. C., Ryan, L. M. & Woodruff, T. J. Dose-response relationship of prenatal mercury exposure and IQ: an integrative analysis of epidemiologic data. *Environ. Health Perspect.* **115**, 609–615 (2007).

4. Rice, G. E., Hammitt, J. K. & Evans, J. S. A probabilistic characterization of the health benefits of reducing methylmercury intake in the United States. *Environ. Sci. Technol.* **44**, 5216–5224 (2010).

5. Giang, A. & Selin, N. E. Benefits of mercury controls for the United States. *Proc. Natl. Acad. Sci. U. S. A.* **113**, 286–291 (2016).

6. Shipp, A. M. *et al*. Determination of a site-specific reference dose for methylmercury for fish-eating populations. *Toxicol. Ind. Health.* **16**, 335–438 (2000).

7. Allen, B. C., Hack, C. E. & Clewell, H. J. Use of Markov Chain Monte Carlo analysis with a physiologically-based pharmaco-kinetic model of methylmercury to estimate exposures in US women of childbearing age. *Risk Anal.* **27**, 947–959 (2007).

8. Stern, A. H. A revised probabilistic estimate of the maternal methyl mercury intake dose corresponding to a measured cord blood mercury concentration. *Environ.*

Health Perspect. **113**, 155–163 (2005).

9. Salonen, J. T. *et al*. Intake of mercury from fish, lipid peroxidation, and the risk of myocardial infarction and coronary, cardiovascular, and any death in eastern Finnish men. *Circulation*. **91**, 645–655 (1995).

10. Rosenberg, L., Kaufman, D. W., Helmrich, S. P. & Shapiro, S. The risk of myocardial infarction after quitting smoking in men under 55 years of age. *N. Engl. J. Med.* **313**, 1511–1514 (1985).

11. Willett, W. C., Hennekens, C. H., Bain, C., Rosner, B. & Speizer, F. E. Cigarette smoking and non-fatal myocardial infarction in women. *Am. J. Epidemiol.* **113**, 575–582 (1981).

12. Dobson, A. J., Alexander, H. M., Heller, R. F. & Lloyd, D. M. How soon after quitting smoking does risk of heart attack decline. *J. Clin. Epidemiol.* **44**, 1247–1253 (1991).

13. Bakhru, A. & Erlinger, T. P. Smoking cessation and cardiovascular disease risk factors: results from the Third National Health and Nutrition Examination Survey. *PLoS. Med.* **2**, e160 (2005).

14. National Health and Family Planning Commission (NHFPC). China Health and Family Planning Statistical Yearbook. Peking Union Medical College Press, Beijing, China (2016).

15. Liang, S., Xu, M., Liu, Z., Suh, S. & Zhang, T. Socioeconomic drivers of mercury emissions in China from 1992 to 2007. *Environ. Sci. Technol.* **47**, 3234–3240 (2013).

16. Liang, S., Zhang, C., Wang, Y., Xu, M. & Liu, W. Virtual atmospheric mercury

emission network in China. *Environ. Sci. Technol.* **48**, 2807–2815 (2014).

17. Chen, L. et al. Trade-induced atmospheric mercury deposition over China and implications for demand-side controls. *Environ. Sci. Technol*. **52**, 2036–2045 (2018).

18. Hui, M. et al. Mercury flows in China and global drivers. *Environ. Sci. Technol.* **51**, 222–231 (2017).

19. Wiedmann, T., Wilting, H. C., Lenzen, M., Lutter, S. & Palm, V. Quo Vadis MRIO? Methodological, data and institutional requirements for multi-region input–output analysis. *Ecol. Econ.* **70**, 1937–1945 (2011).

20. Steenolsen, K. et al. Accounting for value added embodied in trade and consumption: an intercomparison of global multiregional input–output databases. *Econ. Syst. Res.* **28**, 78–94 (2016).

21. Lenzen, M., Wood, R. & Wiedmann, T. Uncertainty analysis for multi-region input–output models – a case study of the UK's carbon footprint. *Econ. Syst. Res.* **22**, 43–63 (2010).

22. Peters, G. P., Davis, S. J. & Andrew, R. A synthesis of carbon in international trade. *Biogeosciences* **9**, 3247–3276 (2012).

23. Zhang, Q. et al. Transboundary health impacts of transported global air pollution and international trade. *Nature* **543**, 705–709 (2017).

24. Lin, J. T., Liu, Z., Zhang, Q. & Liu, H. Modeling uncertainties for tropospheric nitrogen dioxide columns affecting satellite-based inverse modeling of nitrogen oxides emissions. *Atmos. Chem. Phys.* **12**, 12255–12275 (2012).

25. Lin, J. et al. Global climate forcing of aerosols embodied in international trade. *Nat.*

Geosci. **9**, 790–794 (2016).

26. AMAP/UNEP. Technical background report for the global mercury assessment 2013. (Arctic Monitoringand Assessment Programme, Oslo, Norway/UNEP Chemicals Branch, Geneva, Switzerland, 2013).

27. Wu, Q. et al. Temporal trend and spatial distribution of speciated atmospheric mercury emissions in China during 1978–2014. *Environ. Sci. Technol.* **50**, 13428–13435 (2016).

28. Department of Population and Employment Statistics of the National Bureau of Statistics. Sample Survey Data of 1% of National Population. China Statistics Press, Beijing, China (2015).

29. Standardization Administration of the People's Republic of China (SAC). Maximum levels of contaminants in foods (GB 2762–2005). SAC, Beijing, China (2005).

30. Meng, B. et al. The process of methylmercury accumulation in rice (Oryza sativa L.). *Environ. Sci. Technol.* **45**, 2711–2717 (2011).

31. Meng, B. et al. Inorganic mercury accumulation in rice (Oryza sativa L.). *Environ. Sci. Technol.* **31**, 2093–2098 (2012).

32. State Council of the People’s Republic of China (SC). Recent work on soil environmental protection and comprehensive management. Available at http://www.gov.cn/zwgk/2013-01/28/content_2320888.htm (accessed: 20 September 2018) (2013).

33. Liu, M. et al. Increases of total mercury and methylmercury releases from municipal sewage into environment in China and implications. *Environ. Sci. Technol.* **52**, 124–

134 (2017).

34. Vijayaraghavan, K., Levin, L., Parker, L., Yarwood, G. & Streets, D. Response of fish tissue mercury in a freshwater lake to local, regional, and global changes in mercury emissions. *Environ. Toxicol. Chem.* **33**, 1238–1247 (2014).

35. Selin, N. E., Sunderland, E. M., Knightes, C. D. & Mason, R. P. Sources of mercury exposure for U.S. seafood consumers: implications for policy. *Environ. Health Persp.* **118**, 137–143 (2010).

36. Krabbenhoft, D. P. & Sunderland, E. M. Global change and mercury. *Science* **341**, 1457–1458 (2013).

37. Dijkstra, J. A. et al. Experimental and natural warming elevates mercury concentrations in estuarine fish. *PLoS ONE* **8**, e58401 (2013).

38. Zhang, H., Holmes, C. D. & Wu, S. Impacts of changes in climate, land use and land cover on atmospheric mercury. *Atmos. Environ.* **141**, 230–244 (2016).

39. Kumar, A., Wu, S., Huang, Y., Liao, H. & Kaplan, J. O. Mercury from wildfires: global emission inventories and sensitivity to 2000–2050 global change. *Atmos. Environ.* **173**, 6–15 (2018).

40. Angot, H. et al. Chemical cycling and deposition of atmospheric mercury in polar regions: review of recent measurements and comparison with models. *Atmos. Chem. Phys.* **16**, 10735–10763 (2016).

41. Tilman, D. et al. Diversity and productivity in a long-term grassland experiment. *Science* **294**, 843–845 (2001).

42. Eagles-Smith, C. A. et al. Modulators of mercury risk to wildlife and humans in the

context of rapid global change. *Ambio* **47**, 170–197 (2018).

43. Hylander, L. D. et al. Fish mercury increase in Lago Manso, a new hydroelectric reservoir in tropical Brazil. *J. Environ. Manage.* **81**, 155–166 (2006).

44. Willacker, J. J. et al. Reservoirs and water management influence fish mercury concentrations in the western United States and Canada. *Sci. Total Environ.* **568**, 739–748 (2016).

45. Windham-Myers, L. et al. Mercury cycling in agricultural and managed wetlands: a synthesis of methylmercury production, hydrologic export, and bioaccumulation from an integrated field study. *Sci. Total Environ.* **484**, 221–231 (2014).

46. Richardson, D. M. et al. Naturalization and invasion of alien plants: concepts and definitions. *Divers. Distrib.* **6**, 93–107 (2010).

47. Eagles-Smith, C. A., Suchanek, T. H., Colwell, A. E., Anderson, N. L. & Moyle, P. B. Changes in fish diets and food web mercury bioaccumulation induced by an invasive planktivorous fish. *Ecol. Appl.* **18**, 213–226 (2008).

48. Nriagu, J., Basu, N. & Charles, S. Environmental justice: The mercury connection. In Mercury in the environment: Pattern and process, ed. Bank. University of California Press, Berkeley, U.S. (2012).

49. Lauber, T. B., Connelly, N. A., Niederdeppe, J. & Knuth, B. A. Urban anglers in the Great Lakes region: fish consumption patterns, influences, and responses to advisory messages. *Sci. Total Environ.* **590–591**, 495–501 (2017).

50. Hightower, J. M. & Moore, D. Mercury levels in high-end consumers of fish. *Environ. Health Perspect.* **111**, 604–608 (2003).

51. Karimi, R., Silbernagel, S., Fisher, N. S. & Meliker, J. R. Elevated blood Hg at recommended seafood consumption rates in adult seafood consumers. *Int. J. Hyg. Environ. Health* **217**, 758–764 (2014).

52. Awata, H., Linder, S., Mitchell, L. E. & Delclos, G. L. Association of dietary intake and biomarker levels of arsenic, cadmium, lead, and mercury among Asian populations in the United States: NHANES 2011–2012. *Environ. Health Perspect.* **125**, 314–323 (2017).

53. Branco, V. et al. Biomarkers of mercury toxicity: past, present, and future trends. *J. Toxicol. Environ. Health B* **20**, 119–154 (2017).

54. Basu, N., Goodrich, J. M. & Head, J. Ecogenetics of mercury: from genetic polymorphisms and epigenetics to risk assessment and decision-making. *Environ. Toxicol. Chem.* **33**, 1248–1258 (2014).

55. Llop, S. et al. Synergism between exposure to mercury and use of iodine supplements on thyroid hormones in pregnant women. *Environ. Res.* **138**, 298–305 (2015).

56. Bradley, M., Barst, B. & Basu, N. A review of mercury bioavailability in humans and fish. *Int. J. Environ. Res. Public Health* **14**, 169 (2017).

57. Jadán-Piedra, C., Clemente, M. J., Devesa, V. & Vélez, D. Influence of physiological gastrointestinal parameters on the bioaccessibility of mercury and selenium from swordfish. *J. Agric. Food Chem.* **64**, 690–698 (2016).

58. Gadd, G. M. Metals, minerals and microbes: geomicrobiology and bioremediation. *Microbiology* **156**, 609–643 (2010).

59. Madan, J. C., Farzan, S. F., Hibberd, P. L. & Karagas, M. R. Normal neonatal microbiome variation in relation to environmental factors, infection and allergy. *Curr. Opin. Pediatr.* **24**, 753–759 (2012).

60. Rice, D. C. Overview of modifiers of methylmercury neurotoxicity: chemicals, nutrients, and the social environment. *Neurotoxicology* **29**, 761–766 (2008).

61. Bjerregaard, P., Fjordside, S., Hansen, M. G. & Petrova, M. B. Dietary selenium reduces retention of methyl mercury in freshwater fish. *Environ. Sci. Technol.* **45**, 9793–9798 (2011).

62. Eagles-Smith, C. A., Ackerman, J. T., Yee, J. & Adelsbach, T. L. Mercury demethylation in waterbird livers: dose-response thresholds and differences among species. *Environ. Toxicol. Chem.* **28**, 568–577 (2010).

63. Hu, X. F., Laird, B. D. & Chan, H. M. Mercury diminishes the cardiovascular protective effect of omega-3 polyunsaturated fatty acids in the modern diet of Inuit in Canada. *Environ. Res.* **152**, 470–477 (2016).

64. Crowe, W. et al. Mercury as an environmental stimulus in the development of autoimmunity – A systematic review. *Autoimmun. Rev.* **16**, 72–80 (2016).

65. Gardner, R. M., Nyland, J. F. & Silbergeld, E. K. Differential immunotoxic effects of inorganic and organic mercury species in vitro. *Toxicol. Lett.* **198**, 182–190 (2010).
