# Supplementary material for: Trans-provincial health impacts of atmospheric mercury emissions in China
Source: Nat Commun. 2019 Apr 2;10:1484. doi: 10.1038/s41467-019-09080-6 (PMC6445112; doi:10.1038/s41467-019-09080-6)
Supplement: Supplementary file 2 — Description of Additional Supplementary Files [file 41467_2019_9080_MOESM2_ESM.pdf]

Description of Additional Supplementary Files

File Name: Supplementary Data 1
Description: Chinese population in 2015 by provinces, domiciles, ages, and genders

File Name: Supplementary Data 2
Description: Sectoral contributions to national intelligence quotient (IQ) decrements and fatal heart attacks caused by each geographical source

File Name: Supplementary Data 3
Description: Hg-related intelligence quotient (IQ) decrements and fatal heart attacks in each Chinese province and their geographical and sectoral sources

File Name: Supplementary Data 4
Description: Concentrations of THg and MeHg in food products harvested in specific regions and provinces

File Name: Supplementary Data 5
Description: Contributions of geographical sources to national anthropogenic emissions, atmospheric deposition, per-foetus intelligence quotient (IQ) decrements, and fatal heart attacks in China

File Name: Supplementary Data 6
Description: Output values of food products in China

File Name: Supplementary Data 7
Description: Per-capita consumption ($kg \cdot year^{-1} \cdot capita^{-1}$) and per-capita daily intake ($g \cdot d^{-1} \cdot capita^{-1}$) of food products by Chinese population in 2010

File Name: Supplementary Data 8
Description: Intake rates of food products for populations in Chinese provinces

File Name: Supplementary Data 9
Description: All parameters of the China Mercury Risk Source-Tracking Model (CMSTM) with their participation or not in the Monte Carlo simulation

File Name: Supplementary Data 10
Description: Uncertainties in main results of this study
